# Analysis of Immune Landscape Reveals Prognostic Significance of Cytotoxic CD4^+^ T Cells in the Central Region of pMMR CRC

**DOI:** 10.3389/fonc.2021.724232

**Published:** 2021-09-22

**Authors:** Jingwen Qi, Xiaoyan Liu, Peian Yan, Shangwen He, Yuhao Lin, Zhiwei Huang, Shenyan Zhang, Siyu Xie, Yanfeng Li, Xiaofei Lu, Yingjun Wu, Yangshu Zhou, Juanjuan Yuan, Ting Cai, Xiaojun Zheng, Yanqing Ding, Wei Yang

**Affiliations:** ^1^Department of Pathology, School of Basic Medical Sciences, Southern Medical University, Guangzhou, China; ^2^Department of Pathology, Nanfang Hospital, Southern Medical University, Guangzhou, China; ^3^Guangdong Provincial Key Laboratory of Molecular Oncologic Pathology, Southern Medical University, Guangzhou, China; ^4^The First School of Clinical Medicine, Southern Medical University, Guangzhou, China; ^5^Research Department of Medical Sciences, Guangdong Provincial People’s Hospital, Guangdong Academy of Medical Sciences, Guangzhou, China

**Keywords:** mismatch repair-proficient colorectal cancer, multiplex immunohistochemistry, tumor infiltrating lymphocytes, tumor immune microenvironment, neoadjuvant chemotherapy

## Abstract

**Background:**

Mismatch repair proficient colorectal cancer (pMMR CRC) lacks effective treatments and has a poor prognosis, which can be attributed to the complexity of tumor microenvironment. The coordinated function of immune cells is vital to anti-tumor immunity. However, the spatial characteristics of immune cells in the pMMR CRC immune microenvironment and their relationship with clinical prognosis are not fully understood. Meanwhile, the immune modulatory effect of neoadjuvant chemotherapy (NCT), which is the first-line treatment of pMMR CRC, needs further investigation. Therefore, this study aims to explore the spatial dynamics of immune cells and its prognostic value in pMMR CRC.

**Methods:**

We analyzed the various immune cells in formalin-fixed, paraffin-embedded tumor tissues which were collected from 77 patients with stage II/III of pMMR CRC, including 39 non-NCT treated and 38 NCT treated patients. We used the optimized multiplex immunohistochemistry (mIHC) to identify and quantify the density, type and location of immune cells in pMMR CRC. Multivariate survival analysis was performed to assess the relationship of immune profiles and clinical prognosis of pMMR CRC patients.

**Results:**

The densities of most T cell subsets, B cells and macrophages were higher in the central region of the pMMR CRC than in the invasion margin. Tumor infiltrating lymphocytes (TILs), especially the infiltration of CD4^+^ GzmB^+^ T cells in the central region of the tumor was identified to be positively correlated with the prognosis of the patients. Multivariate analysis confirmed that CD4^+^ GzmB^+^ T cells population was an independent predictor of disease-free survival (DFS) in non-NCT group. Meanwhile, NCT enhanced the infiltration of CD4^+^ GzmB^+^ T cells in the central region of the pMMR CRC, which was also identified as an independent protective factor of overall survival (OS) and DFS in NCT group.

**Conclusion:**

We demonstrated that the level of CD4^+^ GzmB^+^ T cells located in the center of tumor could provide great prognostic value for pMMR CRC patients. And the application of neoadjuvant chemotherapy further improves the infiltration of CD4^+^ GzmB^+^ T cells in the central compartment. Further studies into the application of CD4^+^ GzmB^+^ T cells in tumor immunotherapy are needed.

## Introduction

Colorectal cancer (CRC) is the third most common cancer in the world with ever-increasing morbidity and mortality (1, 2). Although great progress has been made in traditional treatments such as surgery, radiotherapy, and chemotherapy, the prognosis of CRC patients remains poor (3). Therefore, the need to find new and effective treatments for CRC is of great importance.

In recent years, immune checkpoint blockade (ICB) therapy has been successfully utilized in the treatment of various solid tumors, including colorectal cancer. However, about 85% of patients with pMMR CRC suffer from low response activity to ICB treatment and poor prognosis. Resistance to immunotherapy can be partly attributed to the lack of immune cells infiltration (4–6). Studies have confirmed that the composition and spatial distribution of immune cells in the tumor microenvironment (TME) greatly influence the efficacy of immunotherapy and patient outcomes (7–11). For example, patients with considerable infiltration of immune cells in tumors, especially CD8^+^ T cells, helper T cells (CD4^+^), and macrophages, would benefit most from the immunotherapy (12, 13). However, the spatial distribution profiles of immune cells in the pMMR CRC immune microenvironment and the relationships with clinical prognosis have not been clearly established, which warrants further investigation.

Chemotherapy is the first-line treatment for advanced CRC. Some previous studies suggest that chemotherapy can impair the anti-tumor immunity by increasing the expression of PD-L1 in non-small cell lung cancer, ovarian cancer, and glioma (14–18). While others suggest that chemotherapy can enforce anti-tumor effect by enhancing immune cells infiltration and changing their localization (19). Therefore, it is important to study the alteration of the distribution of immune cells induced by neoadjuvant chemotherapy in pMMR CRC.

Immunohistochemistry is a widely used method to visualize the immune cell distribution in tissue section. However, it can only detect one type of immune cell in the target tissue, limiting its ability to investigate the spatial distribution of multiple immune cell subgroups in a single slide (20–22). Therefore, multiplex immunohistochemistry (mIHC) has been recently applied in exploring the TME in head and neck tumors, pancreatic cancer, and lung cancer (21–23). This improved method can perform the *in situ* analysis of the spatial distribution characteristics of each subgroup of immune cells. The application of mIHC also allows the investigation into the interplay of tumor cells with immune cells on a single slide, providing a new strategy for in-depth study of the TME.

The aim of this study, was to elaborate the immune profile of pMMR CRC TME and its relationship with patient prognosis using optimized multiple immunohistochemistry technology. Furthermore, the alteration induced by neoadjuvant chemotherapy in the immune profile of pMMR CRC and their clinical significance were analyzed, which may provide new insights on the application of immunotherapy in pMMR CRC patients.

## Materials and Methods

### Patients and Database

Clinical-pathological data and paraffin-embedded specimens were collected from 77 patients who were diagnosed with pMMR CRC and underwent surgery with radical surgical resection in Nanfang Hospital (Guangzhou, China) between March 2011 and July 2018. All patients had no evidence of distant metastases and had not received radiotherapy, targeted therapy, or immunotherapy previously. The clinical and pathological parameters of the cohort groups were obtained from the patient’s medical and pathological records. Among the 77 patients, 39 underwent primary surgical resection (non-Neoadjuant chemotherapy (NCT) group). The comparison group consisted of 38 patients who received NCT prior to surgical resection. The pathological diagnosis was performed according to the World Health Organization (WHO) Classification of Tumors of the digestive System, 5th-Edition (24). The tumor stage was classified according to the American Joint Committee on Cancer/International Union Against Cancer (AJCC/UICC) Staging Manual (8th edition) (25). Clinical and pathological data included age, tumor size, gender, lymphovascular invasion (LVI), perineural invasion (PNI), tumor differentiation, clinical tumor stage (cT), clinical node stage (cN), and clinical Tumor-Node-Metastasis (TNM) staging system (**Table 1**).

**Table 1:** Characteristics of pMMR CRC patients who received neoadjuvant chemotherapy (NCT) or did not receive NCT (non-NCT) (n = 77).

Clinicopathological parameters	Non-NCT (n = 39)	NCT (n = 38)	*P* value
	n (%)	n (%)	
Age (years)			0.307
≤ 60	23 (59)	18 (47.4)	
> 60	16 (41)	20 (52.6)	
Tumor size^a^ (cm)			0.226
≤ 4	26 (66.7)	30 (78.9)	
> 4	13 (33.3)	8 (21.1)	
Gender			0.162
Male	24 (62)	29 (76)	
Female	15 (38)	9 (24)	
LVI^a^			0.591
Negative	31 (79.5)	32 (84.2)	
Positive	8 (20.5)	6 (15.8)	
PNI^a^			0.680
Negative	36 (92.3)	33 (86.8)	
Positive	3 (7.7)	5 (13.2)	
Tumor differentiation^a^			0.209
Poor/Moderate	27 (69.2)	31 (81.6)	
Well	12 (30.8)	7 (18.4)	
cT^b^			0.573
cT3	22 (56.4)	19 (50)	
cT4	17 (43.6)	19 (50)	
cN^b^			0.570
cN0	21 (53.8)	18 (47.4)	
cN+	18 (46.2)	20 (52.6)	
cTNM^b^			0.570
II	21 (53.8)	18 (47.4)	
III	18 (46.2)	20 (52.6)	

a Based on the pathological diagnose.

b Based on the clinical imaging assessments.

LVI, Lymphovascular invasion. PNI, Perineural invasion.

### Multiplex Immunohistochemistry Staining

We established two panels to assess the relationship between spatial distribution and phenotype of tumor infiltrating immune cells. Panel 1 consisted of subtype and functional markers of T cells [CD8α, Granzyme B, CD4, and CD103 (**Table S1**)], and panel 2 consisted of biomarkers for myeloid and B cells [CD66b, CD68, and CD20 (**Table S1**)]. The prepared FFPE tissue slides were deparaffinized in xylene and rehydrated in decreasing concentrations of ethanol (100%, 95%, 90%, 80% and 70%; 2 min each). Then, the rehydrated tissue sections were incubated for 8 min in citrate antigen retrieval solution (pH 6.0), for 20 min in 3% hydrogen peroxide to block endogenous peroxidase activity, and for 30 min in serum-free protein to block solution. Sections were incubated with primary antibody at 4°C overnight, followed with horseradish peroxidase (HRP)-conjugated secondary antibody at room temperature for 1h. Then, 3-amino-9-ethylcarbazole was used to visualize the chromogenic reaction as red staining. The slides were counterstained with hematoxylin and mounted with 70% glycerol, and then an automatic digital slice scanning system (KF-PRO-120) was utilized for whole-slide scanning. After scanning, slide coverslips were removed in hot water (50°C), and tissue sections were destained in 95% ethanol for 10 min. The tissue sections were incubated in citrate antigen retrieval solution (pH 6.0) for antibody stripping and secondary antigen retrieval, according to a modified protocol described previously (26, 27). Sequential staining with subsequent biomarkers was processed similar to the previous rounds of blocking, antibody incubation, chromogenic reaction, and antibody stripping, and acquired images were overlaid to obtain multi-marker images (**Supplementary Figure S1**). Before subsequent immune marker staining, the stripping of primary antibody was confirmed (**Supplementary Figure S2A**). Human tonsil tissue with the primary antibodies was used as a positive control (**Supplementary Figure S2B**).

Automatic digital slide scanning of stained tissue were acquired after each round on the KF whole-slide scanner system (KF-PRO-120) at 400× magnification (ocular 10×, with an objective of 40×). Based on the proposed selection criteria (11), tumor regions of interest (ROIs) were exported using K-viewer software (http://www.kfbio.cn/). To make multicolor images, 3-amino-9-ethylcarbazole and hematoxylin color signals of ROIs were extracted from each single-marker image by color deconvolution, and converted to grayscale. Each immune marker signal was then converted into an indicated pseudo-colored image. Finally, multiple-colored images were merged with Image J, in which different pseudo-colors represent different types of immune cells (**Supplementary Figure S3**).

### Analysis of Tumor-Infiltrating Lymphocytes (TILs) by mIHC

The densities of various IHC-determined immune cell subsets were evaluated separately in indicated regions in the paraffin-embedded specimens, based on the recommendation of the *International TILs Working Group* and adapted with some practical modifications (11). According to the guidelines, tumor areas can be subdivided into two sub-regions: the central tumor (CT) and the invasive margin (IM) (11). Then each region was divided into the stromal and intratumoral region. Therefore, the distribution of TILs in tumor areas was evaluated in four compartments, which were the intratumoral region of the central tumor (intratumoral CT, iCT), the stromal region of the central tumor (stromal CT, sCT), the intratumoral region of the invasive margin (intratumoral IM, iIM) and the stromal region of invasive margin (stromal IM, sIM) (**Supplementary Figure S4**). After each round of whole-slide scanning, three to five ROIs per region were selected on a random basis at high magnification (ocular×10, with an objective of ×20). The average densities of various immune cells in each compartment were calculated as the numbers of positive cells per unit (mm^2^). Moreover, the TILs in tumor compartments with tertiary lymphoid structures, crush artifacts, widespread necrosis and glandular cavity were excluded. The fibroblasts in stroma were identified by their spindle-shaped nuclei; while tumor cells were characterized by enlarged oval nuclei, a high nuclear to cytoplasm ratio and glandular structures. Given the specificity and sensitivity of the CD66b antibody, the infiltration of granulocytes was determined by cell morphology in combination with IHC.

### Statistical Analysis

Clinical data of patients were recorded for analysis. Enumeration data were expressed as a component ratio or rate, and their comparison between groups was conducted by Chi-square test or continuity correction of Chi-square test. The Shapiro-Wilk test was first used to check the normality before comparing continuous data across groups. The Mann–Whitney U test was used to compare differences of independent samples between groups that were not normally distributed, and paired samples were assessed using the paired Wilcoxon rank-sum test. The non-parametric statistical Friedman test analyzed differences of independent samples among three or more groups. According to each subgroup’s median level of immune cell infiltration, they were assigned to a high and low infiltration group. The Kaplan-Meier survival method was adopted to assess the overall survival, followed by a log-rank test to compare differences between groups. Significant indexes with *p* < 0.05 obtained from univariate analysis were introduced into the Cox proportion hazards regression model for multivariate analysis, aiming to identify independent factors influencing the survival rate. IBM SPSS 22.0 and Graph Pad 8.0 were respectively used for statistical processing and figure formatting. A two-sided *p* < 0.05 considered as statistically significant *p < 0.05, **p < 0.01, ***p < 0.001, ****p < 0.0001, and n.s. represent for not significant.

## Results

### Correlation Between the Spatial Distribution of Tumor Infiltrating Immune Cell, Clinicopathological Factors, and the Prognosis of pMMR CRC Patients

Recent studies have revealed that in some cases of pMMR CRC, the type, density, and spatial distribution of infiltrating immune cells in the tumor offer a better prognostic value than TNM staging (28). Based on the TILs evaluation method proposed by Hendry et al. (29), the tumor area of pMMR CRC was first separated into central and marginal areas, both of which were subdivided into intratumoral and stromal regions. Then the density and spatial distribution of immune cells in the main area (CT and IM) and the four subregions (iCT, sCT, iIM, sIM) is systematically examined. Moreover, a Kaplan-Meier survival analysis is performed to assess the relationship between the spatial distribution of immune cells and prognosis.

Tumor infiltrating B cells, which form a substantial part of TME, have a controversial role in anti-tumor immunity (30, 31). B cells were known to mediate the humoral and cellular immunity in the TME (32). And tumor infiltrating B cells were previously recognized as the center of the immune network, which interacted with TILs by presenting antigen and secreting interferon (7, 33, 34). Besides, evidence suggests that the more infiltration of B cells predicted better outcomes in ovarian cancer, cervical cancer and non-small cell lung cancer (NSCLC) (35–38). However, some tumor infiltrating B cell subsets, especially regulatory B cells, exhibited a pro-tumor effect. Our results indicated that compared with the sIM region, CD20^+^ B cells (**Supplementary Figures S5A, B**) were more centralized in the sCT region of tumor. No significant difference in terms of OS and DFS was found between patients with high and low B cell infiltration in the CT (**Tables S2**, **S3**). Macrophages have also been documented to play a dual role in tumor immunity, as they polarized into pro-inflammatory M1 subtype and anti-inflammatory M2 subtype (39). Some reports consistently showed that higher macrophage infiltration was correlated with better survival in colorectal cancer (40, 41). It was found that CD68^+^ macrophages were mainly located in the CT, most obviously in sCT (**Supplementary Figures S5C, D**). But there was no significant correlation between the infiltration of macrophages and survival (**Tables S2**, **S3** **)**. The overall level of granulocytes was generally associated with a poor prognosis (42, 43). It was also reported that tumor infiltrating neutrophils can improve the outcome of CRC patients by enhancing the activation, proliferation and cytokine release of TILs (44). Unlike B cells and macrophages, CD66b^+^ granulocytes were more concentrated in IM, especially in iIM (**Supplementary Figures S5E, F**). Similarly, there was no significant difference of DFS or OS between the low and high granulocytes group **(** **Tables S2**, **S3** **)**.

**Figure 1:**
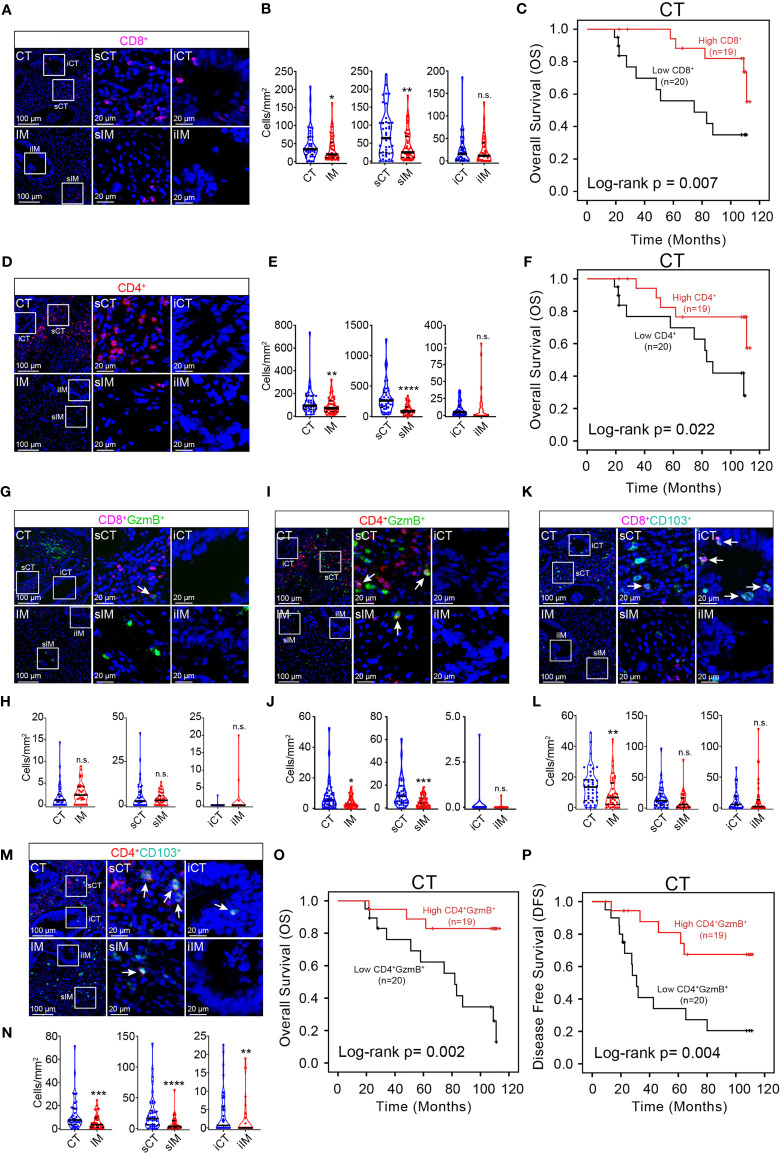
mIHC helps identify immune prognostic markers in pMMR CRC patients. Representative mIHC images of pMMR CRC not treated with NCT show the spatial distribution patterns of CD8^+^ T cells **(A)**, CD4^+^ T cells **(D)** CD8^+^ GzmB^+^ T cells **(G)**, CD4^+^ GzmB^+^ T cells **(I)**, CD8^+^ T_RM_ cells **(K)** and CD4^+^ T_RM_ cells **(M)** in CT and IM region. The violin plots provide statistical comparisons for the density of CD8^+^ T cells **(B)**, CD4^+^ T cells **(E)**, CD8^+^ GzmB^+^ T cells **(H)**, CD4^+^ GzmB^+^ T cells **(J)**, CD8^+^ T_RM_ cells **(L)** and CD4^+^ T_RM_ cells **(N)** in CT and IM region. The thick dashed lines and thin dotted lines denote the median and interquartile range, respectively. Statistical significances were determined *via* Wilcoxon matched-pairs signed rank test, with *P < 0.05, **P < 0.01, ***P < 0.001, ****P < 0.0001, n.s. not significant. Univariate survival analysis of patients was performed to assess the OS and DFS according to the density of CD8^+^ T cells **(C)**, CD4^+^ T cells **(F)**, and CD4^+^ GzmB^+^ T cells **(O, P)** in the CT, using the Kaplan-Meier method.

Since there shows no significant correlation between the density and localization of tumor infiltrating B cells, macrophages, and granulocytes with patient prognosis, the density and subsets of T cells, which are vital to anti-tumor responses, were analyzed in the pMMR CRC TME. It was observed that among major T cell subsets, CD8^+^ T cells (**Figures 1A, B** **)** and CD4^+^ T cells (**Figures 1D, E** **)** were both mainly distributed in CT, especially in sCT; and there was no significant difference in the distribution of CD8^+^ and CD4^+^ T cells in iCT and iIM. Accumulating evidence has suggested that the density of CD8^+^ and CD4^+^ T cells was positively correlated with improved survival (45). Consistently, it is found that the higher density of CD8^+^ and CD4^+^ T cells in the central tumor region was linked to longer OS (**Figures 1C, F** **)**. Then, we analyzed the density of cytotoxic T cell subsets (CD8^+^ GzmB^+^ and CD4^+^ GzmB^+^) and tissue-resident memory T cell subsets (CD8^+^ CD103^+^ and CD4^+^ CD103^+^) in CT and IM. In contrast to CD8^+^ GzmB^+^ T cells which show no unique distribution (**Figures 1G, H** **)**, the infiltration of CD4^+^ GzmB^+^ T cells (**Figures 1I, J**), CD8^+^ CD103^+^ T cells (**Figures 1K, L** **)**, and CD4^+^CD103^+^ T cells (**Figures 1M, N** **)** all increased in CT compared with IM. Moreover, univariate survival analysis indicated that CD4^+^ GzmB^+^ T cells infiltration levels in the central area of the tumor positively correlated with the patient’s OS and DFS (**Figures 1O, P** **)**. The median OS, 3-year and 5-year survival rates of colorectal cancer patients in a high density group of CD8^+^ T cells, CD4^+^ T cells, or CD4^+^ GzmB^+^ T cells were higher than the low-density group respectively **(** **Table S2**). Furthermore, increased infiltration of CD4^+^ GzmB^+^ T cells in the CT region led to prolonged DFS (**Table S3**). In summary, T cells, B cells, and macrophages were more concentrated in the CT region of the pMMR CRC, especially in the sCT region. CD66b^+^ granulocytes were mainly located in the IM region. The density of CD4^+^ GzmB^+^ T cells in the CT region positively related with the prognosis of pMMR CRC patients, which may be a potential prediction of prognosis.

Next, the correlation of different pathological factors and prognosis is assessed. It is found that cTNM negatively correlated with OS and DFS in pMMR CRC patients. No correlation was found between the remaining factors and patient prognosis (**Tables S2**, **S3**).

### Clinicopathological Characteristics of pMMR CRC Patients in Non-NCT Group and NCT Group

77 patients with pMMR CRC were divided into non-NCT and NCT groups according to whether they received neoadjuvant chemotherapy before surgery. The baseline clinicopathological information of the two groups of colorectal cancer patients is presented in **Table 1**. Among them, 38 patients (49%) received neoadjuvant chemotherapy, while the remaining 39 patients (51%) did not. Baseline characteristics were compared between these two groups, including age, tumor size, gender, lymphovascular invasion (LVI), and perineurial invasion (PNI), clinical T stage, clinical N stage, and clinical TNM staging. No statistically significant differences was founded in the baseline metric (*P* > 0.05). Therefore, the two sets of data were comparable.

### Neoadjuvant Chemotherapy Can Promote the Infiltration of CD4^+^ GzmB^+^ T Cells in the Overall Tumor Area

As mentioned above, the infiltration of immune cells in the central region is deeply linked to the survival of pMMR CRC patients. Increasing evidence suggests that chemotherapy could improve the tumor microenvironment by increasing the infiltration of cytotoxic T cell, tissue-resident memory T cells and B cells (46). However, the immunomodulatory effect of neoadjuvant chemotherapy on locally advanced pMMR CRC has not yet been elucidated. Here, we explored the effect of neoadjuvant chemotherapy on the infiltration of immune cells in the overall tumor area of pMMR CRC.

**Figure 2:**
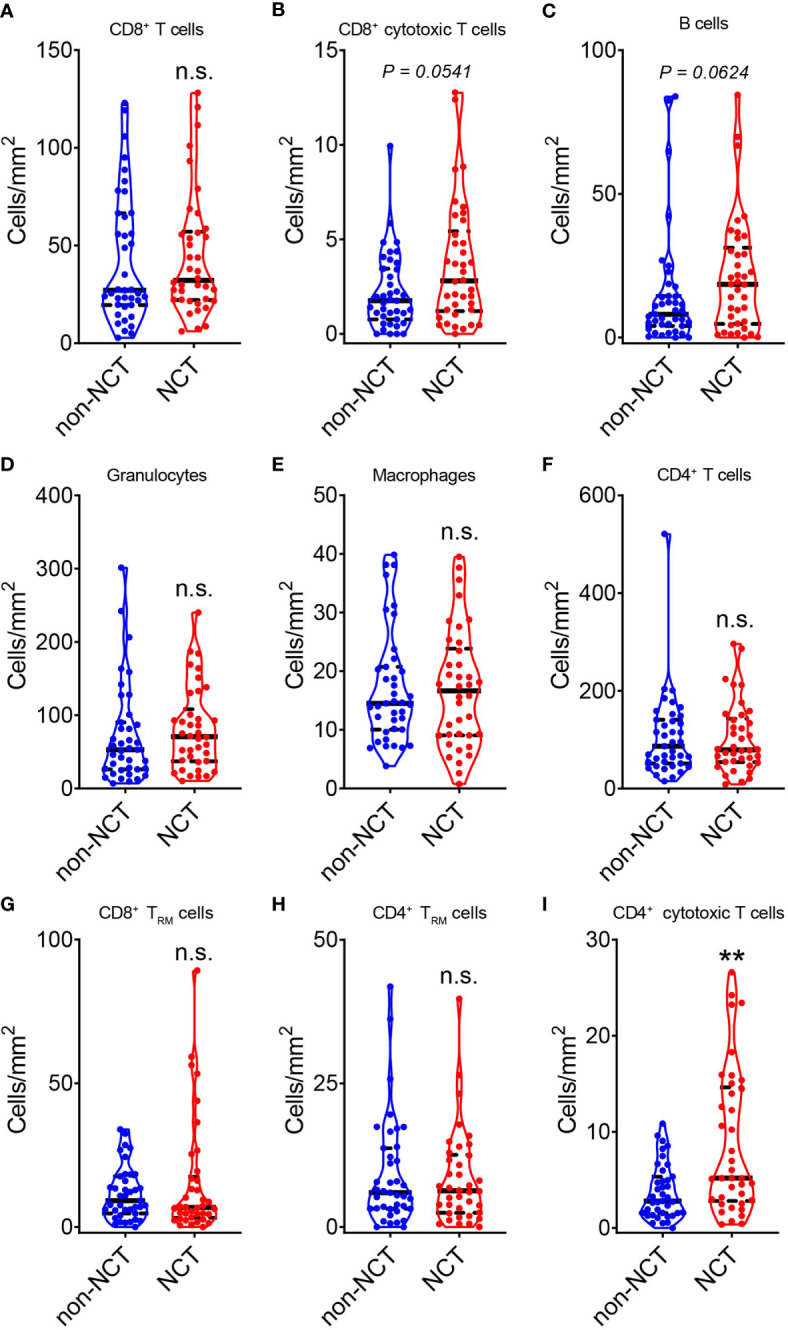
Evaluation of the effect of neoadjuvant chemotherapy on the total TILs in pMMR CRC. The violin plots displayed the alterations in CD8^+^ T cells **(A)**, CD8^+^ GzmB^+^ T cells **(B)**, CD20^+^ B cells **(C)**, CD66b^+^ granulocytes **(D)**, CD68^+^ macrophages **(E)**, CD4^+^ T cells **(F)**, CD8^+^ T_RM_ cells **(G)**, CD4^+^ T_RM_ cells **(H)** and CD4^+^ GzmB^+^ T cells **(I)** during neoadjuvant chemotherapy (NCT). The thick dashed lines and thin dotted lines denote the median and interquartile range, respectively. Statistical significances were determined *via* Mann-Whitney tests, with **P < 0.01, n.s. not significant.

The results showed a slightly increase in the infiltration of CD8^+^ T cells (**Figure 2A**), CD8^+^ GzmB^+^ T cells (**Figure 2B**), B cells (**Figure 2C**), granulocytes (**Figure 2D**), and macrophages (**Figure 2E**) in tumor from NCT group compared with non-NCT group, but there was no significance in these cell subsets. Next, T cell subsets were analyzed. It is found that NCT treatment did not alter the distribution of CD4^+^ T cells (**Figure 2F**), CD8^+^ T_RM_ cells (CD8^+^ CD103^+^) (**Figure 2G**), CD4^+^ T_RM_ cells (CD4^+^ CD103^+^) (**Figure 2H**) and CD4^+^ GzmB^+^ T cells were significantly increased in the overall tumor area after neoadjuvant chemotherapy (**Figure 2I**). Thus, neoadjuvant chemotherapy may only promote the infiltration of immune cells to a certain level in the whole area of pMMR CRC. Moreover, CD4^+^ GzmB^+^ T cells were the main immune cell population that was significantly upregulated after chemotherapy.

### Neoadjuvant Chemotherapy Promotes the Infiltration of CD4^+^ GzmB^+^ T Cells in CT and Correlates With Better Prognosis in pMMR CRC Patients

As illustrated in **Figure 1**, the spatial distribution of immune cells in pMMR CRC is heterogeneous, mainly concentrated in sCT, which suggest the importance of the regional effect in the immune response. However, in most study of the immunomodulatory effect of neoadjuvant chemotherapy, the alternations of distribution of immune cells in different tumor subregions is not well clarified. Therefore, the regional regulatory effect of neoadjuvant chemotherapy on immune cells is analyzed by comparing the changes of immune cells in each tumor subregion before and after neoadjuvant chemotherapy.

Firstly, the changes in the infiltration of immune cells in the invasive margin after chemotherapy was evaluated. It was found that the distribution of immune cells in the IM region were comparable in both groups (**Table S4**). The same results were obtained in the stromal and intratumoral region of IM (**Table S4**). These results suggested neoadjuvant chemotherapy did not alter the immune profile of the IM region.

**Figure 3:**
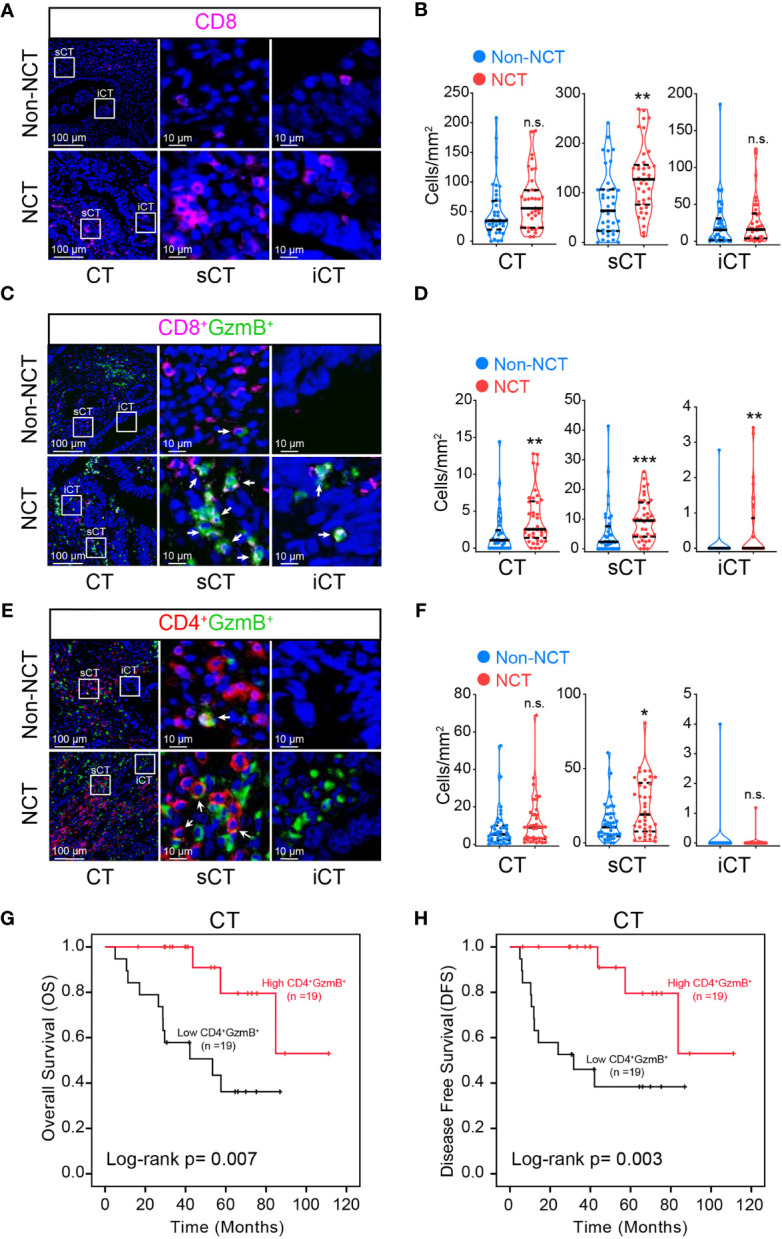
Assessment of the effect of neoadjuvant chemotherapy on T cells in pMMR CRC sub-regions. Representative mIHC images of pMMR CRC show the spatial distribution patterns of CD8^+^ T cells **(A)**, CD8^+^ GzmB^+^ T cells **(C)** and CD4^+^ GzmB^+^ T cells **(E)** in non-NCT group and NCT group. The violin plots provide statistical comparisons for the density of CD8^+^ T cells (**B**), CD8^+^ GzmB^+^ T cells **(D)** and CD4^+^ GzmB^+^ T cells **(F)** in non-NCT group and NCT group. The thick dashed lines and thin dotted lines denote the median and interquartile range, respectively. Statistical significances were determined *via* Mann-Whitney tests, with *P < 0.05, ** *P* < 0.01, *** *P* < 0.001, n.s. not significant. Univariate survival analysis of patients was performed to assess the OS **(G)** and DFS **(H)** according to the density of CD4^+^ GzmB^+^ T cells in the CT, using the Kaplan-Meier method.

Next, the immune profile of the central region of tumor with chemotherapy treatment was assessed. It was found that the infiltration of CD8^+^ T cells in sCT was promoted in NCT group (**Figures 3A, B** **)**. However, the increase of CD8^+^ T cells in sCT of the NCT group did not significantly influence the OS and DFS (**Tables S5**, **S6**). Nonetheless, the density of infiltrating CD8^+^ GzmB^+^ T cells in CT, as well as in its two subregions (iCT and sCT), was enhanced in NCT group (**Figures 3C, D** **)**, but the increased density of CD8^+^ GzmB^+^ T cells in CT had no impact on the overall survival (**Table S5**). The density of CD4^+^ GzmB^+^ T cells in sCT was increased after NCT treatment (**Figures 3E, F** **)**. Further analysis showed that the higher infiltration of CD4^+^ GzmB^+^ T cells in CT was associated with improved OS and DFS (**Figures 3G, H** **)**, which is consistent with our findings in the non-NCT group. These results suggested an immune-boosting effect of chemotherapy in pMMR CRC, which may be mainly dependent the higher infiltration of CD4^+^ GzmB^+^ T cells.

In addition, the changes of macrophages, B cells, and granulocytes in CT post neoadjuvant chemotherapy were analyzed. It is found notable increase in the infiltration level of B cells (**Supplementary Figures S6A, B**) and granulocytes (**Supplementary Figures S6C, D**) in sCT. However, the distribution of macrophages in the sCT and iCT was not altered in NCT group (**Supplementary Figures S6E, F**). Furthermore, the density of macrophages, B cells, and granulocytes in CT of NCT or non-NCT group were not associated with the OS and DFS of pMMR CRC patients respectively (**Tables S5**, **S6**).

The above results indicate that neoadjuvant chemotherapy was spatially heterogeneous in regulating the immune profile in pMMR CRC. Neoadjuvant chemotherapy enhanced recruitment of CD8^+^ T cells, cytotoxic T cells, B cells, and granulocytes in the stroma of central tumor, but not in the invasive margin, which partly justified the small increase of immune infiltration in the overall tumor after chemotherapy. The increased infiltration of CD4^+^ GzmB^+^ T cells in the central tumor area contributed to a better prognosis in the NCT group.

We also examined the predictive value of different clinical and pathological factors, including age, gender, tumor size, tumor differentiation, perineural invasion (PNI) and lymphovascular invasion (LVI). In all these clinicopathological factors, only PNI was significantly and inversely correlated with OS in pMMR CRC patients (*p* = 0.036) (**Table S5**), whereas other clinicopathological factors showed no influence in the patient’s OS and DFS (**Tables S5**, **S6**).

### The Level of CD4^+^ GzmB^+^ T Cell Infiltration in the CT Is an Independent Prognostic Factor in Patients With pMMR CRC

In the above univariate survival analysis, it has been found that the density of infiltrating immune cells in the central tumor was correlated with prognosis. To further identify independent prognostic factors, a multivariate survival analysis based on Cox regression is performed.

In the non-NCT group, the TNM staging system was a significant independent factor for both OS and DFS [hazard ratio (HR), 3.622, and 3.903; *p* = 0.035, and 0.011, respectively, Cox regression; n = 39]. However, high levels of $\text{CD}8_{\text{CT}}^{+},\text{CD}4_{\text{CT}}^{+}$, CD4^+^ $\text{Gzm}{\text{B}}_{\text{CT}}^{+}$ T cells were not significant in the multivariate Cox regression analysis on overall survival, indicating that the infiltration of these immune cells was influenced by TNM staging. However, CD4^+^ $\text{Gzm}{\text{B}}_{\text{CT}}^{+}$ was found to be an independent protective factor of DFS [hazard ratio (HR), 0.216; *p* = 0.005, Cox regression; n = 39].

In the NCT group, multivariate survival analysis displayed that CD4^+^ $\text{Gzm}{\text{B}}_{\text{CT}}^{+}$ was an independent protective factor for OS and DFS in pMMR CRC patients [hazard ratio (HR), 0.218, and 0.180; *p* = 0.021, and 0.009, respectively, Cox regression; n = 38]. However, perineural invasion was not an independent predictor of OS (*p* =0.116).

### Verification of CD4^+^ GzmB^+^ T Cell as an Independent Prognostic Factor in Patients With pMMR CRC

Since the density of CD4^+^ GzmB^+^ T cells may be a promising prediction in pMMR CRC, a verification of the level of CD4^+^ GzmB^+^ T cells in the central tumor in an independent data set is needed. Using the same criteria mentioned in methods, we created a validation set consisting of 100 pMMR CRC patients. The clinicopathological characteristics of the patients in the cohort are listed in **Table 2**. In order to confirm our findings, we quantified the infiltration level of CD4^+^ $\text{Gzm}{\text{B}}_{\text{CT}}^{+}$ T cells was quantified and the patients were divided into two groups based on the median levels of CD4^+^ GzmB^+^ T cells in the central tumor. Then a single variate log-rank test was conducted to analyze the correlation with the clinical factors, the density of CD4^+^ $\text{Gzm}{\text{B}}_{\text{CT}}^{+}$ T cells and survival.

**Table 2:** Clinicopathological parameters of pMMR CRC patients (n = 100).

Clinicopathological parameters	n (%)
Age (years)	
≤ 60	52 (52.0)
> 60	48 (48.0)
Tumor size^a^ (cm)	
≤ 4	61 (61.0)
> 4	39 (39.0)
Gender	
Male	58 (58.0)
Female	42 (42.0)
LVI^a^	
Negative	90 (90.0)
Positive	10 (10.0)
PNI^a^	
Negative	85 (85.0)
Positive	15 (15.0)
Tumor differentiation ^a^	
Poor/Moderate	87 (87.0)
Well	13 (13.0)
cT^b^	
cT1	1 (1.0)
cT2	3 (3.0)
cT3	27 (27.0)
cT4	69 (69.0)
cN^b^	
cN0	50 (50.0)
cN+	50 (50.0)
cTNM^b^	
II	48 (48.0)
III	52 (52.0)

a Based on the pathological diagnose.

b Based on the clinical imaging assessments.

LVI, Lymphovascular invasion. PNI, Perineural invasion.

**Figure 4:**
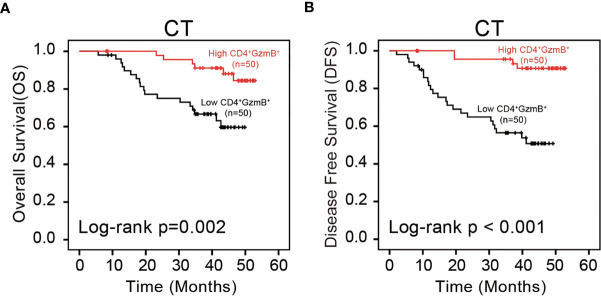
Assessment of the prognostic value of CD4^+^ GzmB^+^ T cells in pMMR CRC. **(A)** Kaplan-Meier curves illustrate the duration of overall survival (OS) and **(B)** disease free survival (DFS) associated with the densities of CD4^+^ GzmB^+^ T cells infiltrated in central tumor region. The red and black lines represent the high-cell-density and low-density.

The relationship between the infiltration level of CD4^+^ $\text{Gzm}{\text{B}}_{\text{CT}}^{+}$ T cells and the overall survival of pMMR CRC patients is analyzed with the Kaplan-Meier and log-rank test. Results showed a positive correlation between the level of CD4^+^ GzmB^+^ T cells in CT and OS (*p = 0.002*) (**Figure 4A**). Patients with higher infiltration of CD4^+^ $\text{Gzm}{\text{B}}_{\text{CT}}^{+}$ T cells had a significantly longer median survival time (50.28 months) and higher 3-year OS rates (84.4%), compared with that of the low-density group (39.25 months, 66.7%) (**Table S7**). Moreover, no obvious correlation was observed between the patient’s OS and clinical factors, including age, sex, tumor size, tumor differentiation, perineural invasion and lymphovascular invasion (*p* > 0.05) (**Table S7**).

Additionally, the influence of CD4^+^ $\text{Gzm}{\text{B}}_{\text{CT}}^{+}$ T cells on DFS was assessed. The level of CD4^+^ $\text{Gzm}{\text{B}}_{\text{CT}}^{+}$ T cells was positively correlated with DFS in the cohort (*p* < 0.001) (**Figure 4B**). The high-density group showed a superior median survival time (50.68 months) and improved 3-year OS rates (96.6%). In contrast, patients with low level of CD4^+^ $\text{Gzm}{\text{B}}_{\text{CT}}^{+}$ T cells were associated with poor outcomes (34.31 months, 56.5%) (**Table S8**). These results also confirmed no significant relationship between the clinicopathological parameters and DFS (**Table S8**).

In brief, the results of the validation set were consistent with our previous results. The infiltration of CD4^+^ GzmB^+^ T cells in the tumor center had a significant prognostic value, positively correlated with good OS and DFS.

## Discussion

In this study, we comprehensively analyzed the complexity of pMMR CRC tumor immune microenvironment, including the composition and spatial distribution of immune cells, as well as their relationship with prognosis. In addition, the differences in the spatial distribution of immune cells in pMMR CRC between the neoadjuvant chemotherapy group and the non-chemotherapy group were firstly elucidated, as well as the relationship between these differences and prognosis and their predictive value. It was found that the intensity of most tumor infiltrating immune cells were the highest in the central region of the tumor. Furthermore, the higher density of CD4^+^, CD8^+^, CD4^+^ GzmB^+^ and CD8^+^ GzmB^+^ T cells positively correlated with longer survival. Besides, this study is the first to use mIHC technology to compare the alterations of immune profile of the non-NCT group and the NCT group in pMMR CRC TME. We found that neoadjuvant chemotherapy promoted the infiltration of CD4^+^ GzmB^+^ T cells, B cells and granulocytes in the central region of the tumor, thus improving the tumor immune microenvironment. Among them, the high density of CD4^+^ GzmB^+^ T cells was a significant and independent factor for a favorable DFS in non-NCT group. Similarly, CD4^+^ GzmB^+^ T cells were identified to be an independent prognostic factor for OS and DFS in post-NCT patients. Together, these results suggested that density of CD4^+^ GzmB^+^ T cells were central to tumor progression and survival, which could be considered as a potential target for immunotherapy.

Nowadays, models based on the quantitative evaluation of the density of immune cells in the center of tumor and the invasive margin of tumor infiltration area, which was first proposed by Galon et al. in 2012 (47), have been successfully developed for a variety of solid tumors (48). In 2018, the prognostic and predictive value of this method was proved to be significantly better than that of TNM staging in colorectal cancer, which was hailed as a revolution in the assessment of CRC patients (49, 50). Based on this method, we observed that most T cell subsets, CD20 ^+^ B cells and CD68^+^ macrophages were mainly distributed in the central region of the tumor, and CD66b^+^ granulocytes were predominately enriched in IM. However, this conclusion is not completely consistent with finding of some previous researches, which have suggested that immune cells are more concentrated in the invasive margin of tumor (51–53). Part of the reason may be the heterogeneity among different types of tumors, which leads to the different spatial distribution characteristics of immune cells (54). Previous studies on the immune microenvironment of colorectal cancer failed to take factors such as tumor MMR status and histological type, which have great impact on the immune cell infiltration, into account. In this study, we only focused on the patients with pMMR CRC. Meanwhile, it is well known that some immune cells, especially B cells, were localized in tertiary lymphoid structures (TLS), which had distinct functions (28, 55). The immune cells in TLS were excluded from our analysis following the guideline published by the international immuno-oncology biomarkers working group in 2017 (29), while some early research did not specify whether TLS was excluded. Another problem is the spatial heterogeneity of TLS, which is localized at the surrounding tissue of tumor and may not be on the same slide of tissue section (56), especially in samples obtained by needle biopsy. Besides, it could be difficult to separate lymphoid aggregates from TLS in the absence of germinal center. Therefore, TLS are excluded in this research, which might also explain why the total number of immune cells in our research is smaller than that in some previous studies.

Recent studies showed that the correlation between tumor infiltrating immune cells in CT and prognosis is stronger than that in IM (57). Consider that the immune cells were mainly distributed in central tumor in our finding, we then focus on the relationship between density of tumor infiltrating immune cells in CT and prognosis. It has found that there was a statistically significant correlation between the infiltration of total CD8^+^ T cells, CD4^+^ T cells in CT and OS (58). Notably, the univariate analysis also identified that a higher density of cytotoxic CD4^+^ T cells in CT, was associated with a better prognosis of pMMR CRC patients. Previous studies have confirmed that CD4^+^ GzmB^+^ T cells were involved in the antitumor immune response of bladder cancer (59), non-small cell lung cancer (60), and colorectal cancer (61). The loss of granzyme B-expressing CD4^+^ T cells predicted poor survival in liver cancer (62). These observations were consistent with our analysis that the DFS and OS of pMMR CRC patients with a higher infiltration level of cytotoxic CD4 ^+^ T cells are longer, which could be proposed as a prognostic biomarker in pMMR CRC and utilize in adoptive cellular therapy (63–66).

As mentioned above, it is known that cytotoxic CD4^+^ GZMB^+^ T cells recognize tumor antigens in an MHC class-II context and lyse tumor cells directly, which can be hampered by tumor infiltrating Treg cells (59, 67, 68). Nonetheless, the details of how CD4^+^ GzmB^+^ T cells located in the central tumor affects the anti-tumor immunity remains unclear and controversial, especially in pMMR CRC. Some argued that cytotoxic CD4^+^ T cells regulated the tumor rejection in a cytolytic molecules-dependent way (e.g., perforin, granzyme B) (69). Others believed that CD4^+^ GZMB^+^ T cells mediated the recruitment of immune cells in melanoma through the release of inflammatory cytokines and chemokines, which would explain the increase infiltration of immune cells in the central tumor (64). It is worth noticing that the underlying anti-tumor mechanism of cytotoxic CD4^+^ GZMB^+^ T cells may be different in pMMR CRC.

Neoadjuvant chemotherapy is known to significantly increase the infiltration of T cells, NK cells, macrophages and other immune cells into solid tumors, which may contribute to its anti-tumor function (70–72). However, we found that neoadjuvant chemotherapy could only slightly promote the infiltration of immune cells in the overall tumor area, among which the infiltration of CD4^+^ GzmB^+^ T cells were significantly increased in NCT group. This could be the result of the sensitivity to chemotherapy in different cancers and more likely, the uneven spatial distribution of immune cells in pMMR CRC. As we expected, neoadjuvant chemotherapy could promote the infiltration of immune cells in CT area, but there was no significant change in the infiltration of immune cells in IM area. As immune cells are more concentrated in CT area, it was speculated that the anti-tumor immune response induced by chemotherapy is preferred in the subregion where most immune cells infiltrate. It turned out that after neoadjuvant chemotherapy, the number of immune cells distributed in CT, especially the sCT subregion showed an increasing trend, among which CD8^+^ T cells, CD8^+^ GzmB^+^ T cells, CD4^+^ GzmB^+^ T cells, and CD66b^+^ granulocytes had significant statistical differences. And we found that the increase of CD4^+^ GzmB^+^ T cells in CT after neoadjuvant chemotherapy led to better OS and DFS, suggesting that CD4^+^ GzmB^+^ cytotoxic T cells may play a protective role in pMMR CRC.

As far as we were concerned, this study is the first to use mIHC technology to compare the changes in the spatial distribution of immune cells in the pMMR CRC immune microenvironment of the non-NCT group and the NCT group. In this research, 3-amino-9-ethylcarbazole (AEC) was used to visualized the cells. Compared with tyramide signal amplification (TSA), chromogenic detection methods based on AEC are more affordable and don’t need additional checks after the heat-mediated stripping cycle. With the help of this technique, we could reveal the immune profile of pMMR CRC in a single section. Also, it allowed us to visualize the communication between different immune cells. Current studies have indicated that the interactions between immune cells were also spatially heterogeneous, which helped to modulate the immune responses. For example, CD20^+^ B cells were more closely correlated with the T cell network in the tumor margin (7). Meanwhile, lymphocytes regulatory network consisting of FoxP3^+^ and Ki67^+^ T cells could be found in the invasive margin of breast cancer (73). Therefore, our future studies should address the interplay of immune cells through direct contact and long-distance signaling in different tumor subregions. Compared with methods based on a single subset of TILs, we believed that models including the spatial characteristic of immune network will have better prognostic values in pMMR CRC.

In general, we discovered that most tumor infiltrating immune cells localized in the central region of the tumor, including CD4^+^ GzmB^+^ T cells. Furthermore, the density of CD4^+^ GzmB^+^ T cells in the central region of the tumor is an independent predictor of prognosis in pMMR CRC patients and can also predict the outcome of patients after neoadjuvant chemotherapy. Our results suggested that CD4^+^ GzmB^+^ T cells could be a potential target for cancer immunotherapy.

## Data Availability Statement

The original contributions presented in the study are included in the article/**Supplementary Material**. Further inquiries can be directed to the corresponding authors.

## Ethics Statement

The studies involving human participants were reviewed and approved by the Ethics Committee of Nanfang Hospital, Nanfang Hospital, Southern Medical University. The patients/participants provided their written informed consent to participate in this study. Written informed consent was obtained from the individual(s) for the publication of any potentially identifiable images or data included in this article.

## Author Contributions

WY, YD, and XZ conceived and designed the project. JQ, SZ, SX, XLu, YZ and YaL collected and assemble data. JQ, XLiu, PY, YW, YuL, ZH performed analysis and interpretation. WY, YD, XZ, JQ, SH, JY, and TC wrote the manuscript. All authors contributed to the article and approved the submitted version.

## Funding

This work was supported by the National Key R&D Program of China (2018YFA0800404 to WY); National Natural Science Foundation of China (81822036 & 31770931 to WY; 81972754 to YD; 31800730 to XZ, Natural Science Foundation of Guangdong Province (2017A030306030 to WY; 2020A1515011246 to XZ), and the China Postdoctoral Science Foundation (2017M622730 to XZ).

## Conflict of Interest

The authors declare that the research was conducted in the absence of any commercial or financial relationships that could be construed as a potential conflict of interest.

## Publisher’s Note

All claims expressed in this article are solely those of the authors and do not necessarily represent those of their affiliated organizations, or those of the publisher, the editors and the reviewers. Any product that may be evaluated in this article, or claim that may be made by its manufacturer, is not guaranteed or endorsed by the publisher.

## Supplementary Material

Supplementary Figure S1 The technical flowchart of multiplex immunohistochemistry. FFPE tissue sections are first preprocessed and incubated with primary antibody. Then, primary antibody is detected by HRP-conjugated secondary antibody. AEC was used as the chromogen. Stained tissue sections were subsequently counterstained, mounted, and scanned. After each scanning procedure, the slide coverslip was removed, followed by AEC chromogen stripping. Finally, tissue sections underwent antigen retrieval and were then incubated in a blocking buffer before the initiation of a new staining cycle.

Supplementary Figure S2 Antibody-stripping validation of mIHC. **(A)** The mIHC procedure was performed in sequential rounds, as detailed in the **Table S1**. The complete removal of antibody between rounds was confirmed *via* detection of reagents and AEC. Scale bar = 100 μm. **(B)** Comparison of standard IHC and mIHC in the detection of CD4 (left) and CD103 (right) in human tonsil or CRC tissue quantified in five regions with 20× magnification. No significant difference was observed in the detection of CD4^+^ cells (round 3) and CD103^+^ cells (round 4) between standard IHC (round 1) and mIHC. Statistical significance was determined by paired t-test. Scale bar = 100 μm.

Supplementary Figure S3 Digital slice scanning and image processing. Digital scans representing bright-field sequential IHC of one FFPE section of pMMR CRC tissue enable assessment of 4 lymphoid biomarkers. AEC color signals were extracted from each digitized single-marker image by color deconvolution. Bright-field images were inverted, and pseudo-coloring was performed.

Supplementary Figure S4 Regional division strategy for CRC. Immunohistochemistry of CD8^+^ T cells is used to map the region of the tumor. Schematic division of the central region of the tumor (CT) and the infiltrating marginal region of the tumor (IM) at low magnification (left). Scale bar = 400 μm. Images of selected CT and IM areas within the tumor region at low magnification (right) The yellow dotted line represents the stromal region of the tumor, namely stromal region of the central tumor (sCT) and the stromal region of invasive margin (sIM). The area depicted by the blue dotted line is the tumor epithelial region, the intratumoral region of the central tumor (iCT) and intratumoral region of the invasive margin (iIM). Scale bar = 25 μm.

Supplementary Figure S5 mIHC helps characterize the tumor immune microenvironment in pMMR CRC. Representative mIHC images of non-NCT group pMMR CRC show the spatial distribution patterns of CD20^+^ B cells **(A)**, CD68^+^ macrophages **(C)**, CD66b^+^ Granulocytes **(E)**, CD8^+^ GzmB^+^ T cells **(G)**, CD8^+^ T_RM_ cells **(I)** and CD4^+^ T_RM_ cells **(K)** in CT and IM region. The violin plots provide statistical comparisons for CD20^+^ B cells **(B)**, CD68^+^ macrophages **(D)**, CD66b^+^ Granulocytes **(F)** in CT and IM region. The thick dashed lines and thin dotted lines denote the median and interquartile range, respectively. Statistical significances were determined *via* Wilcoxon matched-pairs signed rank test, with **P* < 0.05, ****P* < 0.001, n.s. not significant.

Supplementary Figure S6 Spatial distribution of B-cell and myeloid cells between non-NCT group and NCT group. Representative mIHC images showing densities of B cells **(A)**, CD66b^+^ Granulocytes **(C)** and CD68^+^ macrophages **(E)** in the CT region of pMMR CRC treated with neoaduvant chemotherapy (NCT) or not treated with NCT (non-NCT). The violin plots provide statistical comparisons for B cells **(B)**, CD66b^+^ Granulocytes **(D)** and CD68^+^ macrophages **(F)** in non-NCT group and NCT group. The thick dashed lines and thin dotted lines denote the median and interquartile range, respectively. Statistical significances were determined *via* Mann-Whitney tests, with **P* < 0.05, ** *P* < 0.01, n.s. not significant.
