# Supplementary figures and images for: Analysis of Immune Landscape Reveals Prognostic Significance of Cytotoxic CD4+ T Cells in the Central Region of pMMR CRC
Source: Front Oncol. 2021 Sep 22;11:724232. doi: 10.3389/fonc.2021.724232 (PMC8493090; doi:10.3389/fonc.2021.724232)

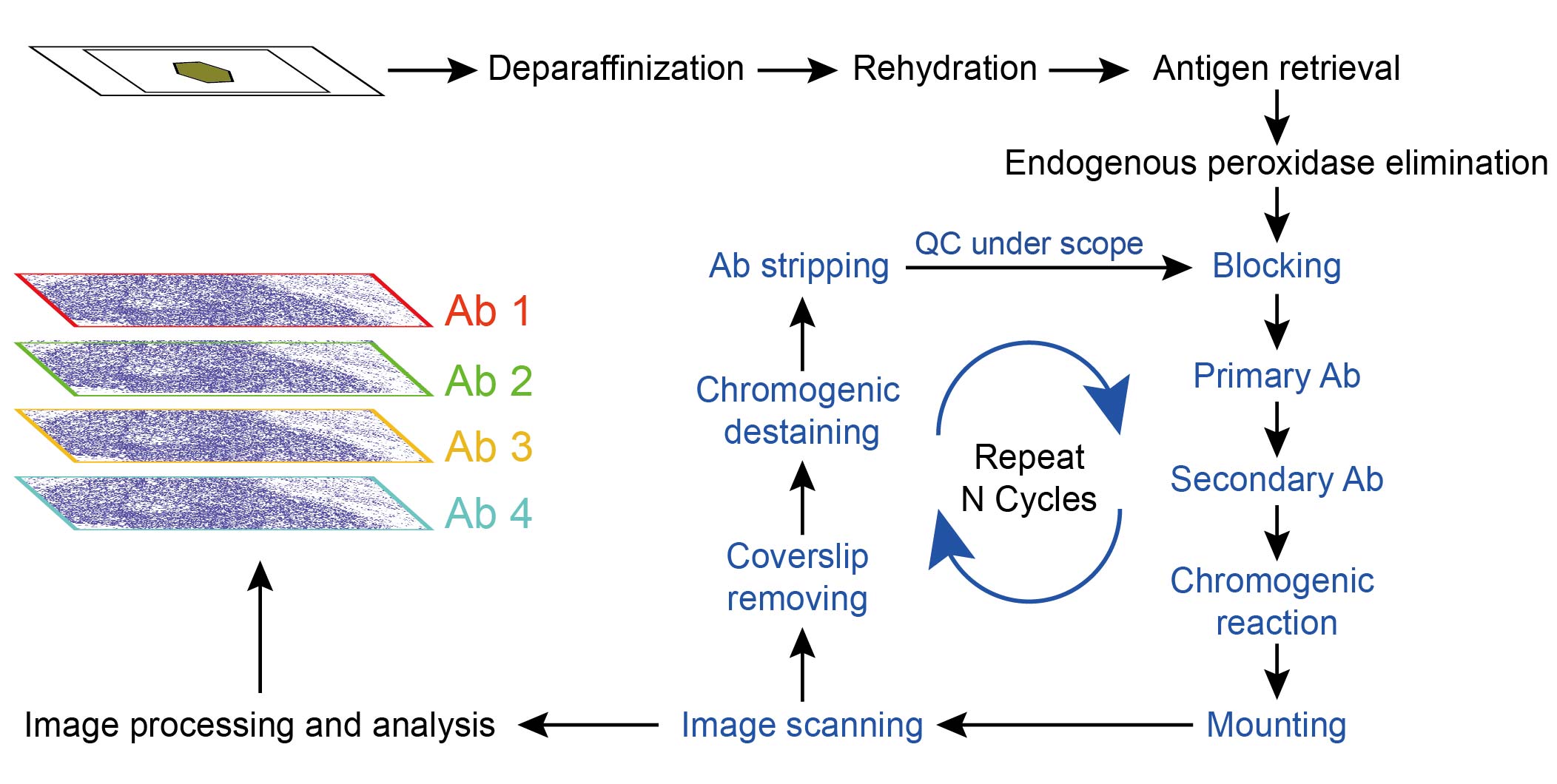

Supplement: Supplementary Figure S1 — The technical flowchart of multiplex immunohistochemistry. FFPE tissue sections are first preprocessed and incubated with primary antibody. Then, primary antibody is detected by HRP-conjugated secondary antibody. AEC was used as the chromogen. Stained tissue sections were subsequently counterstained, mounted, and scanned. After each scanning procedure, the slide coverslip was removed, followed by AEC chromogen stripping. Finally, tissue sections underwent antigen retrieval and were then incubated in a blocking buffer before the initiation of a new staining cycle. [file Image_1.jpeg]

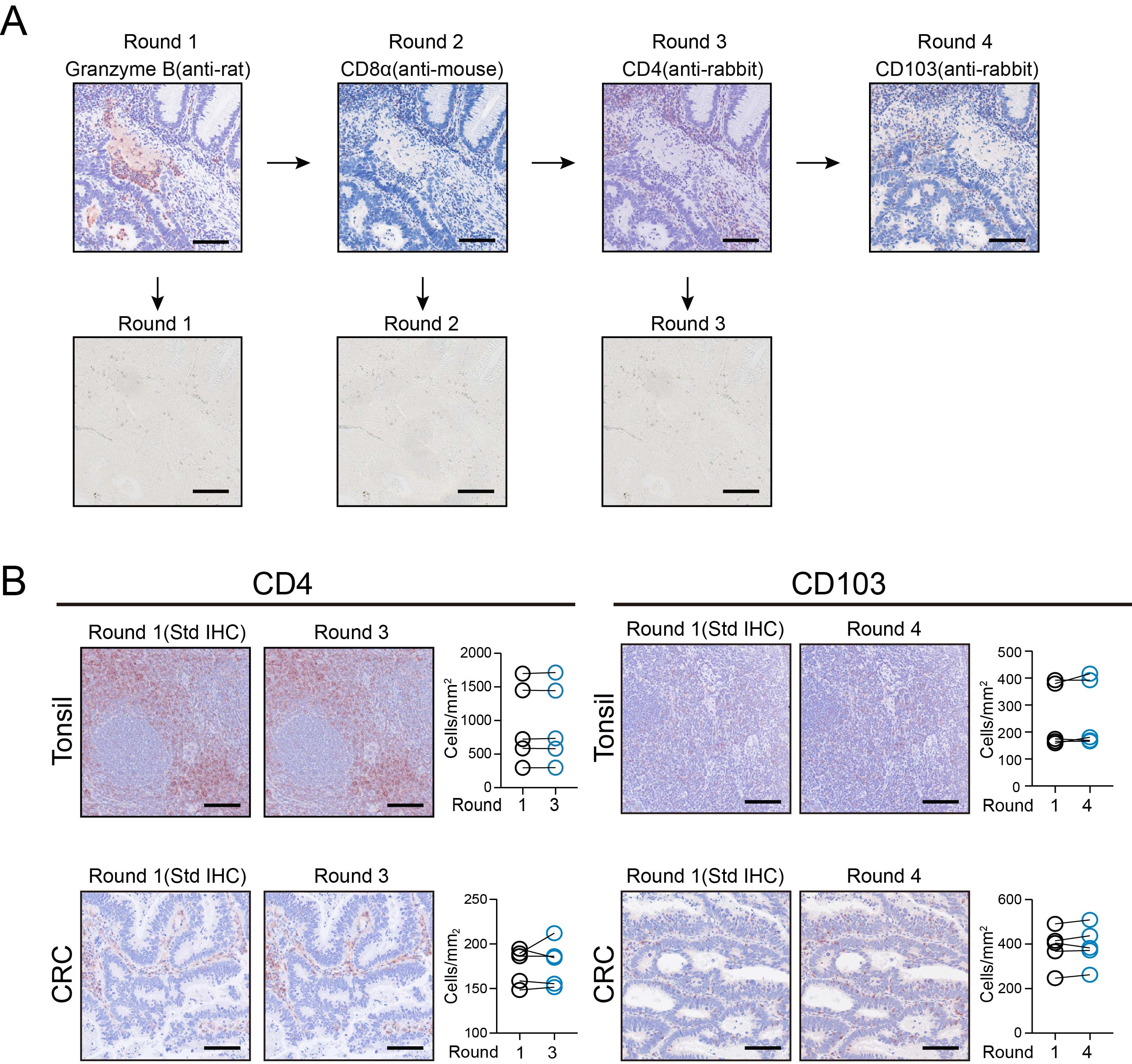

Supplement: Supplementary Figure S2 — Antibody-stripping validation of mIHC. (A) The mIHC procedure was performed in sequential rounds, as detailed in the Table S1. The complete removal of antibody between rounds was confirmed via detection of reagents and AEC. Scale bar = 100 μm. (B) Comparison of standard IHC and mIHC in the detection of CD4 (left) and CD103 (right) in human tonsil or CRC tissue quantified in five regions with 20× magnification. No significant difference was observed in the detection of CD4+ cells (round 3) and CD103+ cells (round 4) between standard IHC (round 1) and mIHC. Statistical significance was determined by paired t-test. Scale bar = 100 μm. [file Image_2.jpeg]

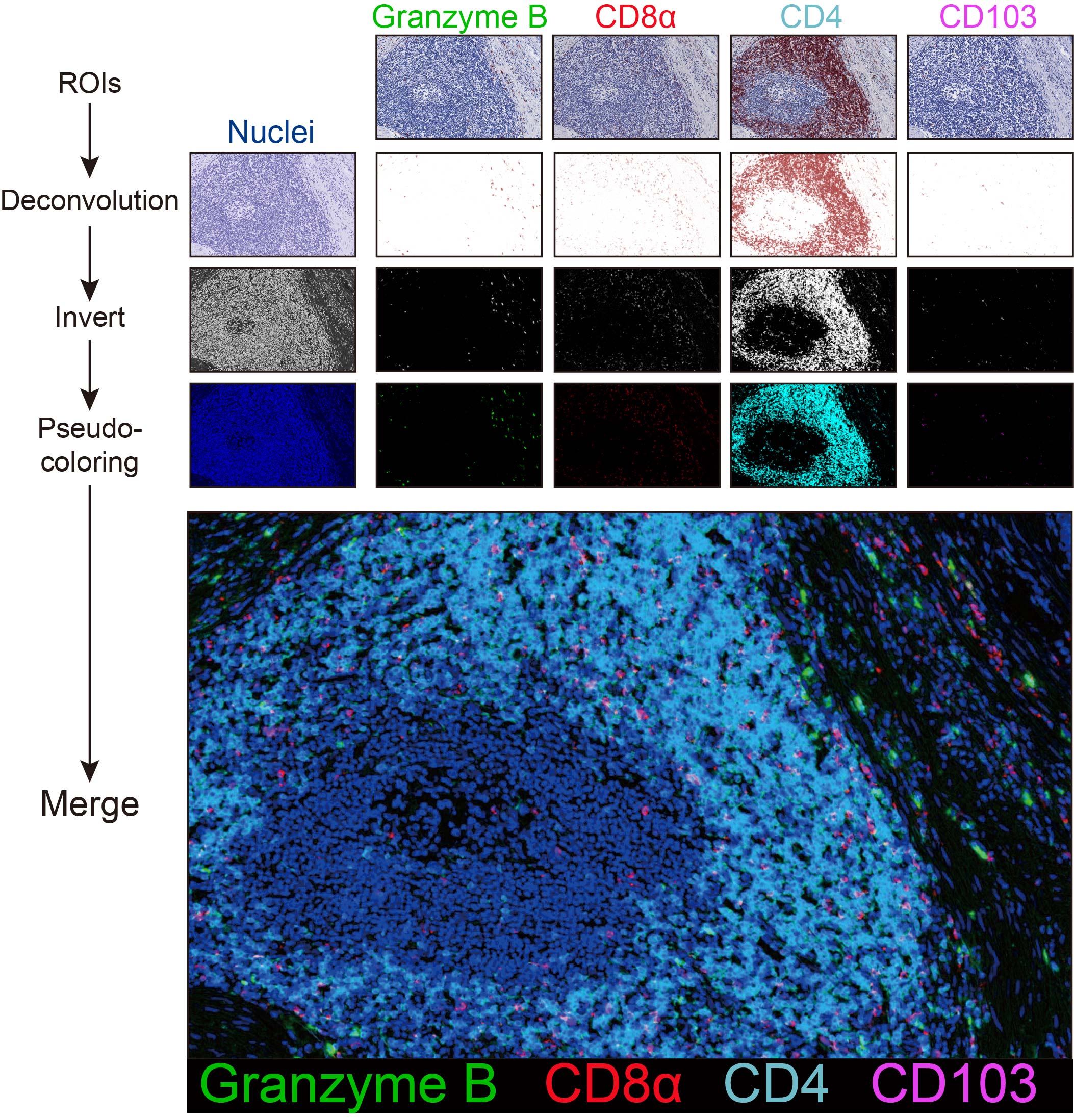

Supplement: Supplementary Figure S3 — Digital slice scanning and image processing. Digital scans representing bright-field sequential IHC of one FFPE section of pMMR CRC tissue enable assessment of 4 lymphoid biomarkers. AEC color signals were extracted from each digitized single-marker image by color deconvolution. Bright-field images were inverted, and pseudo-coloring was performed. [file Image_3.jpeg]

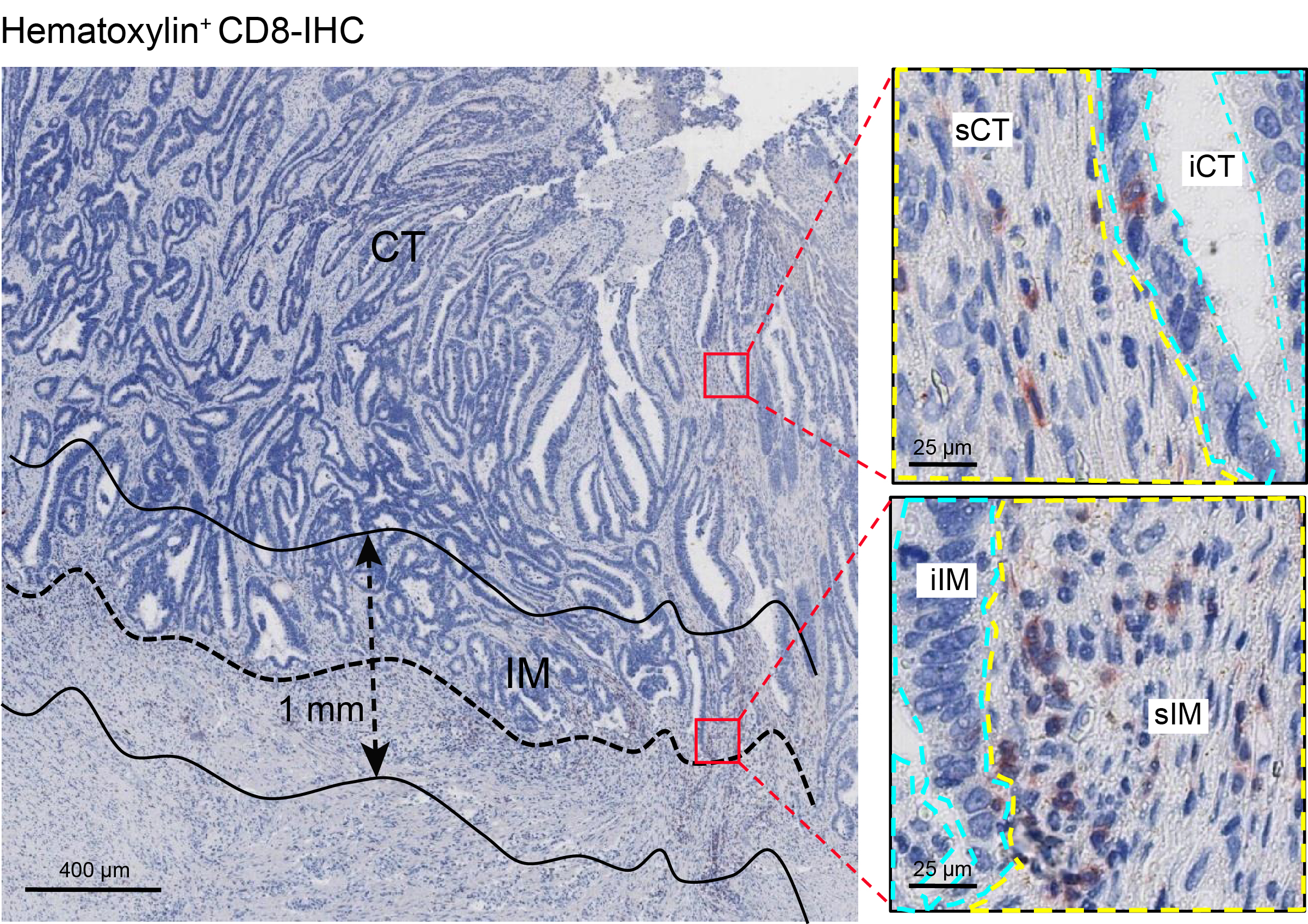

Supplement: Supplementary Figure S4 — Regional division strategy for CRC. Immunohistochemistry of CD8+ T cells is used to map the region of the tumor. Schematic division of the central region of the tumor (CT) and the infiltrating marginal region of the tumor (IM) at low magnification (left). Scale bar = 400 μm. Images of selected CT and IM areas within the tumor region at low magnification (right) The yellow dotted line represents the stromal region of the tumor, namely stromal region of the central tumor (sCT) and the stromal region of invasive margin (sIM). The area depicted by the blue dotted line is the tumor epithelial region, the intratumoral region of the central tumor (iCT) and intratumoral region of the invasive margin (iIM). Scale bar = 25 μm. [file Image_4.jpeg]

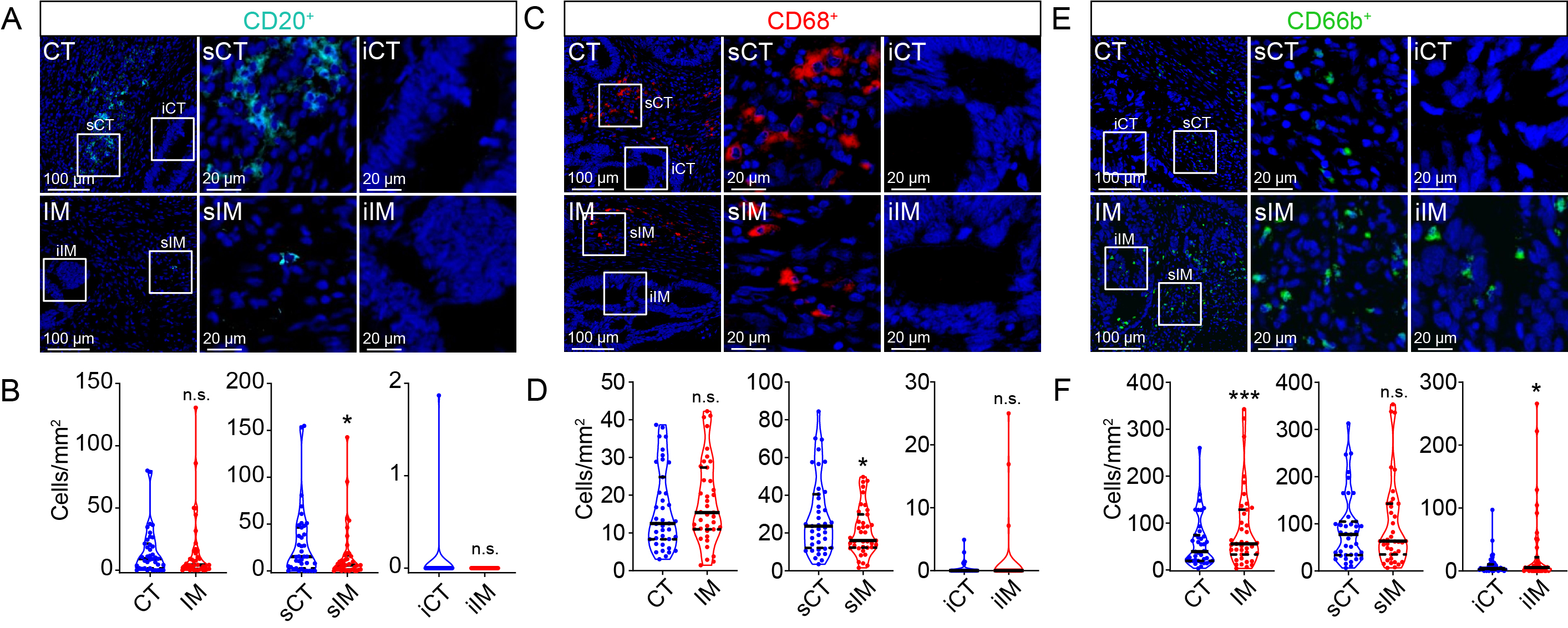

Supplement: Supplementary Figure S5 — mIHC helps characterize the tumor immune microenvironment in pMMR CRC. Representative mIHC images of non-NCT group pMMR CRC show the spatial distribution patterns of CD20+ B cells (A), CD68+ macrophages (C), CD66b+ Granulocytes (E), CD8+ GzmB+ T cells (G), CD8+ TRM cells (I) and CD4+ TRM cells (K) in CT and IM region. The violin plots provide statistical comparisons for CD20+ B cells (B), CD68+ macrophages (D), CD66b+ Granulocytes (F) in CT and IM region. The thick dashed lines and thin dotted lines denote the median and interquartile range, respectively. Statistical significances were determined via Wilcoxon matched-pairs signed rank test, with *P < 0.05, ***P < 0.001, n.s. not significant. [file Image_5.jpeg]

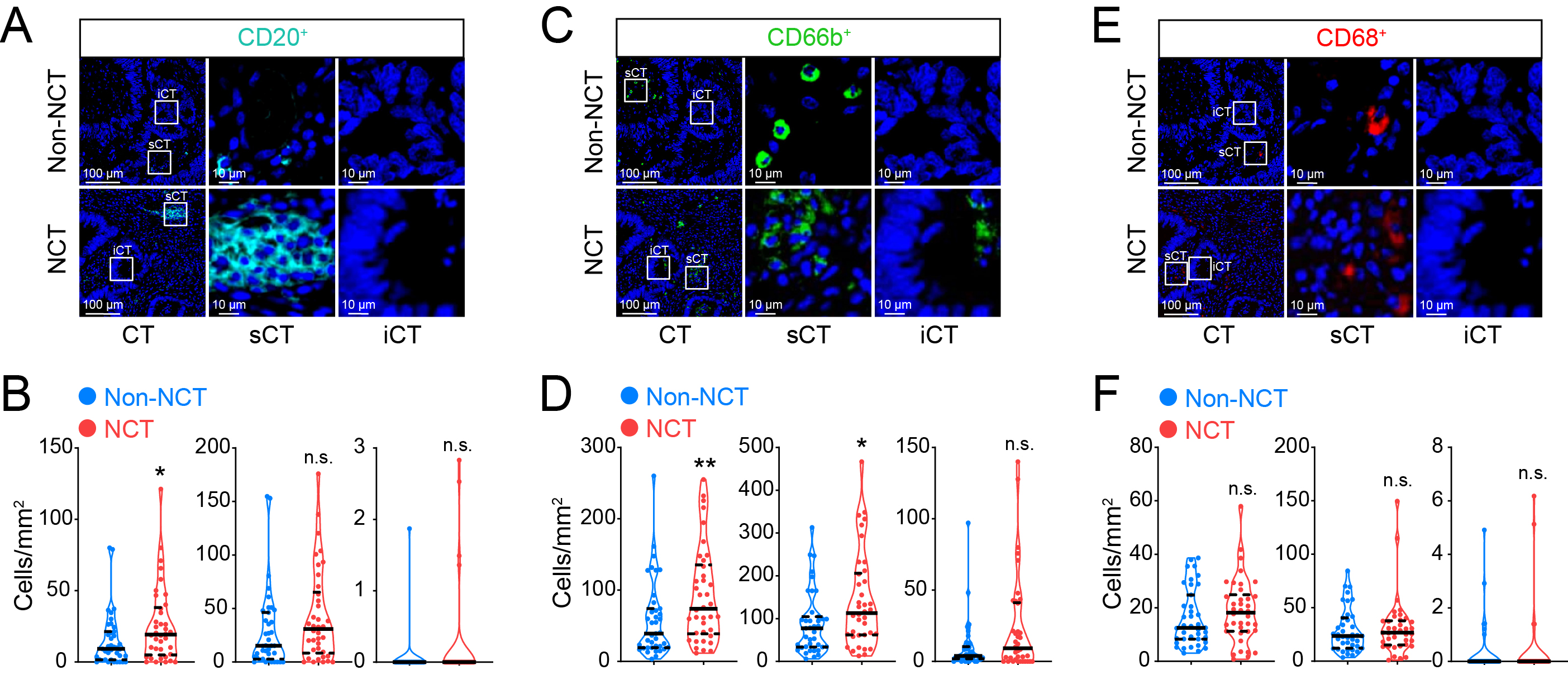

Supplement: Supplementary Figure S6 — Spatial distribution of B-cell and myeloid cells between non-NCT group and NCT group. Representative mIHC images showing densities of B cells (A), CD66b+ Granulocytes (C) and CD68+ macrophages (E) in the CT region of pMMR CRC treated with neoaduvant chemotherapy (NCT) or not treated with NCT (non-NCT). The violin plots provide statistical comparisons for B cells (B), CD66b+ Granulocytes (D) and CD68+ macrophages (F) in non-NCT group and NCT group. The thick dashed lines and thin dotted lines denote the median and interquartile range, respectively. Statistical significances were determined via Mann-Whitney tests, with *P < 0.05, ** P < 0.01, n.s. not significant. [file Image_6.jpeg]
