# Supplementary material for: Analysis of Immune Landscape Reveals Prognostic Significance of Cytotoxic CD4+ T Cells in the Central Region of pMMR CRC
Source: Front Oncol. 2021 Sep 22;11:724232. doi: 10.3389/fonc.2021.724232 (PMC8493090; doi:10.3389/fonc.2021.724232)
Supplement: Supplementary file 7 [file Table_1.docx]

**Table S1 Sequential mIHC panel information**

**Panel 1 Subtype and functional biomarkers of T cells**

| **Sequence** | **Round 1** | **Round2** | **Round 3** | **Round 4** |
| --- | --- | --- | --- | --- |
| **Primary Ab** | Granzyme B | CD8a | CD4 | CD103 |
| **Supplier** | eBioscience | eBioscience | Abcam | Abcam |
| **Product#** | 14-8889-82 | 14-0085-82 | 133616 | 129202 |
| **Concentration** | 1:200 | 1:100 | 1:250 | 1:500 |
| **Species reactivity** | Anti-rat | Anti-mouse | Anti-rabbit | Anti-rabbit |

**Panel 2 Myeloid and B cells biomarkers**

| **Sequence** | **Round 1** | **Round2** | **Round 3** |
| --- | --- | --- | --- |
| **Primary Ab** | CD66b | CD20 | CD68 |
| **Supplier** | Biolegend | eBioscience | Biolegend |
| **Product#** | 305102 | 14-0202-82 | 916104 |
| **Concentration** | 1:50 | 1:500 | 1:2500 |
| **Species reactivity** | Anti-mouse | Anti-mouse | Anti-mouse |
