# Supplementary material for: Analysis of Immune Landscape Reveals Prognostic Significance of Cytotoxic CD4+ T Cells in the Central Region of pMMR CRC
Source: Front Oncol. 2021 Sep 22;11:724232. doi: 10.3389/fonc.2021.724232 (PMC8493090; doi:10.3389/fonc.2021.724232)
Supplement: Supplementary file 8 [file Table_2.docx]

**Table S2. Univariate analysis of factors associated with overall survival (OS) for non-NCT pMMR CRC**

| Variables | 1. year OS   (%) | 5-year OS  (%) | Median OS  （months） | Log rank-X^2^ | *P* value |
| --- | --- | --- | --- | --- | --- |
| Age (years) |  |  |  | 0.123 | 0.725 |
| ≤ 60 | 81.3 | 81.3 | 111 |  |  |
| > 60 | 92.9 | 71.4 | 87.5 |  |  |
| Tumor size (cm) |  |  |  | 0.430 | 0.512 |
| ≤ 4 | 84.1 | 75.7 | 111 |  |  |
| > 4 | 91.7 | 78.6 | 92 |  |  |
| Gender |  |  |  | 0.355 | 0.551 |
| Male | 92.3 | 84.6 | 95.9 |  |  |
| Female | 82.0 | 71 | 83.3 |  |  |
| LVI |  |  |  | 3.405 | 0.065 |
| Negative | 89.7 | 82.2 | 111 |  |  |
| Positive | 65.6 | 43.8 | 48.1 |  |  |
| PNI |  |  |  | 3.793 | 0.051 |
| Negative | 87.9 | 78.1 | 111 |  |  |
| Positive | 66.7 | 66.7 | 20 |  |  |
| Tumor differentiation |  |  |  | 0.032 | 0.858 |
| Poor / Moderate | 83.8 | 70.6 | 111 |  |  |
| Well | 91.7 | 91.7 | 109 |  |  |
| cTNM |  |  |  | 5.710 | **0.017** |
| Ⅱ | 100 | 93.3 | 103.2 |  |  |
| Ⅲ | 71.8 | 59.8 | 83.2 |  |  |
| CD8_CT_ |  |  |  | 7.213 | **0.007** |
| Low | 69.8 | 55.8 | 74.5 |  |  |
| High | 100 | 94.1 | 104.3 |  |  |
| CD4_CT_ |  |  |  | 5.270 | **0.022** |
| Low | 76.8 | 69.8 | 83.2 |  |  |
| High | 94.1 | 82.4 | 97.7 |  |  |
| CD8GzmB_CT_ |  |  |  | 1.424 | 0.233 |
| Low | 78.3 | 78.3 | 109 |  |  |
| High | 94.7 | 74.4 | 92.5 |  |  |
| CD4GzmB_CT_ |  |  |  | 9.173 | **0.002** |
| Low | 83 | 62.3 | 82 |  |  |
| High | 94.7 | 88.8 | 101.6 |  |  |
| CD8CD103_CT_ |  |  |  | 0.452 | 0.501 |
| Low | 79.4 | 68.8 | 82.3 |  |  |
| High | 94.7 | 86.8 | 111 |  |  |
| CD4CD103_CT_ |  |  |  | 0.006 | 0.939 |
| Low | 88.9 | 83.3 | 111 |  |  |
| High | 82.8 | 67.7 | 84 |  |  |
| CD20 _CT_ |  |  |  | 0.165 | 0.684 |
| Low | 83.5 | 65.6 | 85.8 |  |  |
| High | 88 | 88 | 109 |  |  |
| CD66b _CT_ |  |  |  | 0.106 | 0.745 |
| Low | 84.1 | 72.9 | 111 |  |  |
| High | 87.4 | 80.2 | 91.1 |  |  |
| CD68CT _CT_ |  |  |  | 2.341 | 0.126 |
| Low | 84.1 | 77.1 | 87.5 |  |  |
| High | 88.2 | 76.5 | 92.1 |  |  |
