# Supplementary material for: Analysis of Immune Landscape Reveals Prognostic Significance of Cytotoxic CD4+ T Cells in the Central Region of pMMR CRC
Source: Front Oncol. 2021 Sep 22;11:724232. doi: 10.3389/fonc.2021.724232 (PMC8493090; doi:10.3389/fonc.2021.724232)
Supplement: Supplementary file 9 [file Table_3.docx]

**Table S3 Univariate analysis of factors associated with disease free survival (DFS) for non-NCT pMMR CRC**

| Variables | 3-year DFS  (%) | 5-year DFS  (%) | Median DFS  （months） | Logrank-X^2^ | *P* value |
| --- | --- | --- | --- | --- | --- |
| Age (years) |  |  |  | 0.241 | 0.623 |
| ≤ 60 | 66.1 | 60.6 | 64 |  |  |
| > 60 | 61.3 | 52.5 | 65.2 |  |  |
| Tumor size (cm) |  |  |  | 0.026 | 0.872 |
| ≤ 4 | 62.2 | 53.3 | 61.6 |  |  |
| > 4 | 70.5 | 70.5 | 80 |  |  |
| Gender |  |  |  | 0.589 | 0.443 |
| Male | 58.3 | 58.6 | 80 |  |  |
| Female | 69.2 | 57.7 | 64 |  |  |
| LVI |  |  |  | 1.817 | 0.178 |
| Negative | 66.9 | 62.7 | 80 |  |  |
| Positive | 52.5 | 35 | 46.1 |  |  |
| PNI |  |  |  | 0.873 | 0.35 |
| Negative | 65.3 | 58.4 | 65.2 |  |  |
| Positive | 66.7 | 66.7 | 19.2 |  |  |
| Tumor differentiation |  |  |  | 1.298 | 0.255 |
| Poor / Moderate | 57.3 | 47.7 | 46.1 |  |  |
| Well | 80.2 | 80.2 | 82.7 |  |  |
| cTNM |  |  |  |  |  |
| II | 87.7 | 87.7 | 90.1 | 6.322 | **0.012** |
| III | 44.4 | 33.3 | 32 |  |  |
| CD8_CT_ |  |  |  | 3.266 | 0.071 |
| Low | 54.4 | 46.7 | 46.1 |  |  |
| High | 75.3 | 69 | 81.4 |  |  |
| CD4_CT_ |  |  |  | 2.001 | 0.157 |
| Low | 49.3 | 49.3 | 32 |  |  |
| High | 77 | 65.1 | 78.1 |  |  |
| CD8GzmB_CT_ |  |  |  | 0.441 | 0.507 |
| Low | 57.1 | 51.4 | 65.2 |  |  |
| High | 73.3 | 65.2 | 64 |  |  |
| CD4GzmB_CT_ |  |  |  | 8.378 | **0.004** |
| Low | 40.9 | 34.1 | 30.9 |  |  |
| High | 87.7 | 81 | 89.9 |  |  |
| CD8CD103_CT_ |  |  |  | 0.065 | 0.799 |
| Low | 64.3 | 58.9 | 65.2 |  |  |
| High | 60.6 | 51.9 | 61.6 |  |  |
| CD4CD103_CT_ |  |  |  | 0.301 | 0.583 |
| Low | 59.6 | 53.7 | 64 |  |  |
| High | 71.1 | 63.2 | 74.9 |  |  |
| CD20 _CT_ |  |  |  | 0.604 | 0.437 |
| Low | 73.8 | 67.1 | 76.2 |  |  |
| High | 54.7 | 47.9 | 42.6 |  |  |
| CD66b _CT_ |  |  |  | 0.709 | 0.4 |
| Low | 61.9 | 55.7 | 61.6 |  |  |
| High | 66.7 | 59.3 | 75 |  |  |
| CD68CT _CT_ |  |  |  | 1.926 | 0.165 |
| Low | 57.1 | 49.9 | 42.6 |  |  |
| High | 70.5 | 64.1 | 77.9 |  |  |
