# Supplementary material for: Analysis of Immune Landscape Reveals Prognostic Significance of Cytotoxic CD4+ T Cells in the Central Region of pMMR CRC
Source: Front Oncol. 2021 Sep 22;11:724232. doi: 10.3389/fonc.2021.724232 (PMC8493090; doi:10.3389/fonc.2021.724232)
Supplement: Supplementary file 10 [file Table_4.docx]

**Table S4 Comparison of the densities of TILs in IM between the non-NCT and NCT pMMR CRC patients (n=77)**

| Immune cell subsets | IM | | *P* Value | sIM | | *P* Value | iIM | | *P* Value |
| --- | --- | --- | --- | --- | --- | --- | --- | --- | --- |
|  | non-NCT | NCT |  | non-NCT | NCT |  | non-NCT | NCT |  |
|  | Median cell density(cells/mm^2^) | |  | Median cell density(cells/mm^2^) | |  | Median cell density(cells/mm^2^) | |  |
| Panel 1 |  |  |  |  |  |  |  |  |  |
| CD8^+^ | 19.91 | 28.11 | 0.7825 | 24.70 | 37.03 | 0.3275 | 11.44 | 5.23 | 0.1934 |
| CD4^+^ | 62.80 | 60.97 | 0.6375 | 87.19 | 97.45 | 0.9496 | 0.00 | 0.00 | 0.3791 |
| CD8+GzmB^+^ | 2.23 | 1.95 | 0.9411 | 2.78 | 3.10 | 0.7543 | 0.00 | 0.00 | 0.4345 |
| CD4+GzmB^+^ | 2.53 | 2.97 | 0.6823 | 4.53 | 5.47 | 0.1486 | 0.00 | 0.00 | 0.1761 |
| CD8+CD103^+^ | 6.56 | 4.40 | 0.6103 | 5.59 | 5.02 | 0.7358 | 2.25 | 0.51 | 0.5146 |
| CD4+CD103^+^ | 3.48 | 4.08 | 0.913 | 3.64 | 8.93 | 0.2532 | 0.00 | 0.00 | 0.4327 |
|  |  |  |  |  |  |  |  |  |  |
| Panel 2 |  |  |  |  |  |  |  |  |  |
| CD20^+^ | 4.23 | 12.44 | 0.1378 | 6.15 | 13.95 | 0.1618 | 0.00 | 0.00 | 0.1153 |
| CD66b^+^ | 55.58 | 57.25 | 0.6159 | 63.20 | 69.24 | 0.6088 | 5.30 | 6.77 | 0.7805 |
| CD68^+^ | 18.22 | 15.38 | 0.2505 | 16.03 | 16.52 | 0.3532 | 0.00 | 0.00 | 0.8049 |

IM, invasive margin; sIM, stromal IM; iIM, intratumoral IM.
