# Supplementary material for: Analysis of Immune Landscape Reveals Prognostic Significance of Cytotoxic CD4+ T Cells in the Central Region of pMMR CRC
Source: Front Oncol. 2021 Sep 22;11:724232. doi: 10.3389/fonc.2021.724232 (PMC8493090; doi:10.3389/fonc.2021.724232)
Supplement: Supplementary file 11 [file Table_5.docx]

**Table S5 Univariate analysis of factors associated with overall survival (OS) for NCT pMMR CRC**

| Variables | 1. year OS   (%) | 5-year OS  (%) | Median OS  （months） | Log rank-X^2^ | *P* value |
| --- | --- | --- | --- | --- | --- |
| Age (years) |  |  |  | 0.773 | 0.379 |
| ≤ 60 | 66.7 | 57.1 | 84.8 |  |  |
| > 60 | 89.7 | 55.1 | 68.5 |  |  |
| Tumor size (cm) |  |  |  | 0.001 | 0.978 |
| ≤ 4 | 76.0 | 59.5 | 84.8 |  |  |
| > 4 | 87.5 | 43.8 | 57.6 |  |  |
| Gender |  |  |  | 0.709 | 0.400 |
| Male | 50.8 | 50.8 | 66.8 |  |  |
| Female | 86.1 | 56.9 | 84.8 |  |  |
| LVI |  |  |  | 0.660 | 0.417 |
| Negative | 74.4 | 55.4 | 84.8 |  |  |
| Positive | 100 | 50.0 | 57.3 |  |  |
| PNI |  |  |  | 4.391 | **0.036** |
| Negative | 84.4 | 60.3 | 84.8 |  |  |
| Positive | 40.0 | 40.0 | 28.8 |  |  |
| Tumor differentiation |  |  |  | 1.912 | 0.167 |
| Poor / Moderate | 73.4 | 51.1 | 84.8 |  |  |
| Well | 100.0 | 80.0 | 97.6 |  |  |
| cTNM |  |  |  | 0.633 | 0.426 |
| Ⅱ | 83.3 | 66.7 | 84.8 |  |  |
| Ⅲ | 74.1 | 43.9 | 57.6 |  |  |
| CD8_CT_ |  |  |  | 2.479 | 0.115 |
| Low | 72.4 | 45.3 | 57.3 |  |  |
| High | 84.2 | 66.3 | 86.0 |  |  |
| CD4_CT_ |  |  |  | 1.509 | 0.219 |
| Low | 68.4 | 45.6 | 57.6 |  |  |
| High | 88.9 | 66.9 | 84.8 |  |  |
| CD8GzmB_CT_ |  |  |  | 1.512 | 0.219 |
| Low | 72.3 | 41.3 | 57.3 |  |  |
| High | 84.2 | 68.0 | 84.8 |  |  |
| CD4GzmB_CT_ |  |  |  | 7.258 | **0.007** |
| Low | 57.9 | 36.2 | 53.4 |  |  |
| High | 100.0 | 79.5 | 91.9 |  |  |
| CD8CD103_CT_ |  |  |  | 1.826 | 0.177 |
| Low | 72.7 | 49.8 | 57.6 |  |  |
| High | 83.9 | 63.9 | 85.8 |  |  |
| CD4CD103_CT_ |  |  |  | 0.338 | 0.561 |
| Low | 78.9 | 48.4 | 57.6 |  |  |
| High | 78.0 | 66.9 | 84.8 |  |  |
| CD20 _CT_ |  |  |  | 0.910 | 0.340 |
| Low | 78.3 | 71.8 | 84.8 |  |  |
| High | 78.6 | 33.7 | 57.3 |  |  |
| Granulocytes _CT_ |  |  |  | 0.146 | 0.703 |
| Low | 73.7 | 60.3 | 84.8 |  |  |
| High | 83.6 | 48.8 | 57.6 |  |  |
| CD68CT _CT_ |  |  |  | 0.383 | 0.536 |
| Low | 78.9 | 48.4 | 57.6 |  |  |
| High | 67.1 | 76.5 | 60.5 |  |  |
