# Supplementary material for: Analysis of Immune Landscape Reveals Prognostic Significance of Cytotoxic CD4+ T Cells in the Central Region of pMMR CRC
Source: Front Oncol. 2021 Sep 22;11:724232. doi: 10.3389/fonc.2021.724232 (PMC8493090; doi:10.3389/fonc.2021.724232)
Supplement: Supplementary file 12 [file Table_6.docx]

**Table S6 Univariate analysis of factors associated with disease free survival (DFS) for NCT pMMR CRC**

| Variables | 3-year DFS  (%) | 5-year DFS  (%) | Median DFS  （months） | Log rank-X^2^ | *P* value |
| --- | --- | --- | --- | --- | --- |
| Age (years) |  |  |  | 0.880 | 0.348 |
| ≤ 60 | 65.2 | 55.9 | 83.6 |  |  |
| > 60 | 78.7 | 61.2 | 66.3 |  |  |
| Tumor size (cm) |  |  |  | 0.000 | 0.991 |
| ≤ 4 | 71.5 | 60.4 | 83.6 |  |  |
| > 4 | 75.0 | 50.0 | 42.1 |  |  |
| Gender |  |  |  | 0.517 | 0.472 |
| Male | 55.6 | 55.6 | 66.5 |  |  |
| Female | 78.2 | 60.4 | 83.6 |  |  |
| LVI |  |  |  | 0.929 | 0.335 |
| Negative | 67.3 | 57.7 | 83.6 |  |  |
| Positive | 100.0 | 50.0 | 57.3 |  |  |
| PNI |  |  |  | 3.112 | 0.078 |
| Negative | 77.4 | 62.5 | 83.6 |  |  |
| Positive | 40.0 | 40.0 | 14.2 |  |  |
| Tumor differentiation |  |  |  | 1.806 | 0.179 |
| Poor / Moderate | 66.7 | 55.0 | 83.6 |  |  |
| Well | 100.0 | 80.20 | 97.6 |  |  |
| cTNM |  |  |  | 0.685 | 0.408 |
| Ⅱ | 76.9 | 68.4 | 83.6 |  |  |
| Ⅲ | 68.4 | 47.9 | 57.3 |  |  |
| CD8_CT_ |  |  |  | 1.654 | 0.198 |
| Low | 73.7 | 46.1 | 57.3 |  |  |
| High | 72.3 | 72.3 | 84.8 |  |  |
| CD4_CT_ |  |  |  | 1.951 | 0.163 |
| Low | 55.3 | 55.3 | 53.9 |  |  |
| High | 88.5 | 66.6 | 80.6 |  |  |
| CD8GzmB_CT_ |  |  |  | 0.740 | 0.390 |
| Low | 73.3 | 41.9 | 57.3 |  |  |
| High | 71.5 | 71.5 | 83.6 |  |  |
| CD4GzmB_CT_ |  |  |  | 8.550 | **0.003** |
| Low | 46.1 | 38.4 | 31.7 |  |  |
| High | 100.0 | 79.5 | 91.6 |  |  |
| CD8CD103_CT_ |  |  |  | 1.723 | 0.189 |
| Low | 68.4 | 53.2 | 54.1 |  |  |
| High | 76.6 | 65.7 | 83.7 |  |  |
| CD4CD103_CT_ |  |  |  | 0.479 | 0.489 |
| Low | 67.5 | 52.5 | 56.5 |  |  |
| High | 77.6 | 66.5 | 77.2 |  |  |
| CD20 _CT_ |  |  |  | 1.131 | 0.288 |
| Low | 78.9 | 72.4 | 83.6 |  |  |
| High | 64.4 | 38.6 | 57.3 |  |  |
| Granulocytes _CT_ |  |  |  | 0.145 | 0.703 |
| Low | 68.0 | 61.2 | 59.4 |  |  |
| High | 77.6 | 53.2 | 74.6 |  |  |
| CD68CT _CT_ |  |  |  | 0.486 | 0.486 |
| Low | 67.5 | 52.5 | 66.7 |  |  |
| High | 78.3 | 67.1 | 58.9 |  |  |
