# Supplementary material for: Analysis of Immune Landscape Reveals Prognostic Significance of Cytotoxic CD4+ T Cells in the Central Region of pMMR CRC
Source: Front Oncol. 2021 Sep 22;11:724232. doi: 10.3389/fonc.2021.724232 (PMC8493090; doi:10.3389/fonc.2021.724232)
Supplement: Supplementary file 13 [file Table_7.docx]

**Table S7 Univariate analysis of factors associated with overall survival (OS) for pMMR CRC patients.**

| Variables | 3-year OS  （%） | Median OS  （months） | Log rank-X^2^ | *P* value |
| --- | --- | --- | --- | --- |
| Age (years) |  |  | 0.061 | 0.805 |
| ≤ 60 | 76.6 | 45.43 |  |  |
| > 60 | 80.4 | 45.37 |  |  |
| Tumor size (cm) |  |  | 0.719 | 0.396 |
| ≤ 4 | 81.1 | 46.21 |  |  |
| > 4 | 74.3 | 44.39 |  |  |
| Gender |  |  | 0.269 | 0.604 |
| Male | 73.7 | 44.65 |  |  |
| Female | 81.9 | 46.03 |  |  |
| LVI |  |  | 0.002 | 0.968 |
| Negative | 78.6 | 45.62 |  |  |
| Positive | 77.8 | 45.34 |  |  |
| PNI |  |  | 0.005 | 0.944 |
| Negative | 77.8 | 45.47 |  |  |
| Positive | 83.3 | 43.41 |  |  |
| Tumor differentiation |  |  | 0.543 | 0.461 |
| Poor / Moderate | 77.8 | 45.11 |  |  |
| Well | 83.3 | 48.27 |  |  |
| cTNM |  |  | 2.295 | 0.130 |
| II | 89.0 | 48.52 |  |  |
| III | 68.7 | 42.46 |  |  |
| CD4^+^GzmB^+^_CT_ |  |  | 9.725 | **0.002** |
| Low | 66.7 | 39.35 |  |  |
| High | 84.4 | 50.28 |  |  |
