# Supplementary material for: Analysis of Immune Landscape Reveals Prognostic Significance of Cytotoxic CD4+ T Cells in the Central Region of pMMR CRC
Source: Front Oncol. 2021 Sep 22;11:724232. doi: 10.3389/fonc.2021.724232 (PMC8493090; doi:10.3389/fonc.2021.724232)
Supplement: Supplementary file 14 [file Table_8.docx]

**Table S8 Univariate analysis of factors associated with disease free survival (DFS) for pMMR CRC patients.**

| Variables | 3-year DFS  （%） | Median DFS  （months） | Log rank-X^2^ | *P* value |
| --- | --- | --- | --- | --- |
| Age (years) |  |  | 0.295 | 0.587 |
| ≤ 60 | 72.6 | 42.38 |  |  |
| > 60 | 78.4 | 43.86 |  |  |
| Tumor size (cm) |  |  | 1.367 | 0.242 |
| ≤ 4 | 77.8 | 44.39 |  |  |
| > 4 | 71.6 | 41.35 |  |  |
| Gender |  |  | 0.201 | 0.654 |
| Male | 74.3 | 41.94 |  |  |
| Female | 76.4 | 44.08 |  |  |
| LVI |  |  | 0.052 | 0.820 |
| Negative | 75.3 | 43.22 |  |  |
| Positive | 77.8 | 43.89 |  |  |
| PNI |  |  | 0.045 | 0.832 |
| Negative | 75.5 | 43.26 |  |  |
| Positive | 75.0 | 42.45 |  |  |
| Tumor differentiation |  |  | 0.854 | 0.355 |
| Poor / Moderate | 74.4 | 42.77 |  |  |
| Well | 83.3 | 46.46 |  |  |
| cTNM |  |  |  |  |
| II | 84.7 | 46.99 | 3.783 | 0.052 |
| III | 66.9 | 39.42 |  |  |
| CD4^+^GzmB^+^_CT_ |  |  | 18.782 | **＜0.001** |
| Low | 56.5 | 34.31 |  |  |
| High | 96.6 | 50.68 |  |  |
